# Genome Wide Association Study (GWAS) of Chagas Cardiomyopathy in *Trypanosoma cruzi* Seropositive Subjects

**DOI:** 10.1371/journal.pone.0079629

**Published:** 2013-11-20

**Authors:** Xutao Deng, Ester C. Sabino, Edecio Cunha-Neto, Antonio L. Ribeiro, Barbara Ianni, Charles Mady, Michael P. Busch, Mark Seielstad, the REDSII Chagas study group from the NHLBI Retrovirus Epidemiology Donor Study-II (REDS-II), International Component

**Affiliations:** 1 Blood Systems Research Institute, San Francisco, California, United States of America; 2 Department of Infectious Disease/Institute of Tropical Medicine, University of São Paulo, São Paulo, Brazil; 3 Laboratory of Immunology, Heart Institute (InCor)/Division of Clinical Immunology and Allergy, University of São Paulo, São Paulo, Brazil; 4 Institute for Investigation in Immunology/Instituto Nacional de Ciência e Tecnologia, São Paulo, Brazil; 5 Hospital das Clínicas and Faculdade de Medicina, Universidade Federal de Minas Gerais, Belo Horizonte, Brazil; 6 Cardiomyopathy Unit of the Heart Institute (InCor) University of São Paulo Medical School, São Paulo, Brazil; 7 Department of Laboratory Medicine, University of California San Francisco, San Francisco, California, United States of America; 8 Institute for Human Genetics, University of California San Francisco, San Francisco, California, United States of America; Albert Einstein College of Medicine, United States of America

## Abstract

**Background:**

Familial aggregation of Chagas cardiac disease in *T. cruzi*–infected persons suggests that human genetic variation may be an important determinant of disease progression.

**Objective:**

To perform a GWAS using a well-characterized cohort to detect single nucleotide polymorphisms (SNPs) and genes associated with cardiac outcomes.

**Methods:**

A retrospective cohort study was developed by the NHLBI REDS-II program in Brazil. Samples were collected from 499 *T. cruzi* seropositive blood donors who had donated between1996 and 2002, and 101 patients with clinically diagnosed Chagas cardiomyopathy. In 2008–2010, all subjects underwent a complete medical examination. After genotype calling, quality control filtering with exclusion of 20 cases, and imputation of 1,000 genomes variants; association analysis was performed for 7 cardiac and parasite related traits, adjusting for population stratification.

**Results:**

The cohort showed a wide range of African, European, and modest Native American admixture proportions, consistent with the recent history of Brazil. No SNPs were found to be highly (P<10^−8^) associated with cardiomyopathy. The two mostly highly associated SNPs for cardiomyopathy (rs4149018 and rs12582717; P-values <10^−6^) are located on Chromosome 12p12.2 in the SLCO1B1 gene, a solute carrier family member. We identified 44 additional genic SNPs associated with six traits at P-value <10^-6^: Ejection Fraction, PR, QRS, QT intervals, antibody levels by EIA, and parasitemia by PCR.

**Conclusion:**

This GWAS identified suggestive SNPs that may impact the risk of progression to cardiomyopathy. Although this Chagas cohort is the largest examined by GWAS to date, (580 subjects), moderate sample size may explain in part the limited number of significant SNP variants. Enlarging the current sample through expanded cohorts and meta-analyses, and targeted studies of candidate genes, will be required to confirm and extend the results reported here. Future studies should also include exposed seronegative controls to investigate genetic associations with susceptibility or resitance to *T. cruzi* infection and non-Chagas cardiomathy.

## Introduction

Chagas disease is caused by *T. cruzi*, a parasite which is naturally transmitted through several species of haematophagous reduviid bugs. The infection/disease is prevalent in most Latin American countries, where approximately 10 million people are infected with *T. cruzi* and at least 120 million are at risk [1], [2]. In the recent decades, migration from endemic areas brought hundreds of thousands of *T. cruzi*-infected patients to the USA and Europe, turning Chagas disease into a global health concern [1], [2]. Most untreated acute cases evolve into the so-called indeterminate stage of chronic Chagas disease (seropositive but no evidence of the cardiac or digestive forms of the disease as evaluated by clinical examination, ECG and X ray studies) [2].

The progression from the indeterminate to “clinical” stage of chronic Chagas disease, i.e., cardiomyopathy and the mega-syndromes, generally occurs 10 to 20 years after acute infection at a rate of approximately 1–2% per year [3]. Chronic Chagas cardiomyopathy (CCC), the most clinically relevant manifestation of human Chagas disease, affects 20–40% of patients in the chronic phase of the disease, and manifests as heart failure, arrhythmia, heart block, thromboembolism, stroke, and sudden death [2]. These abnormalities generally occur in combination and CCC is characterized by its severity, as well as by a worse prognosis when compared with other cardiomyopathies [2], [4].

The pathogenesis of chronic CCC is not completely understood. There is evidence to indicate that persistent parasitism of heart tissue induces T cell-mediated inflammation, which has been implicated in myocardial tissue damage [5], [6]. In addition, there is evidence to suggest that auto-immunity may contribute significantly to the inflammatory damage to heart cells and the conduction system [6], [7].

The genetic basis for differential rates of *T. cruzi* infection and disease progression was evaluated by Williams-Blangero and colleagues who studied pedigrees from a highly endemic region in the State of Goias, Brazil [8]. They showed that half of the variability in susceptibility to infection (presence of antibody to *T. cruzi*) could be attributed to genetic factors. They also showed that ECG measurements altered by CCC such as PR, QRS and QT intervals were influenced by genetic variation. Using a similar approach, Silva-Grecco et al. showed similar evidence of familial aggregation for *T. cruzi* seropositivity in the city of Agua Comprida, Minas Gerais, Brazil [9].

A number of candidate gene association studies have been performed in Chagas disease, comparing polymorphism frequencies in patients with CCC and asymptomatic carriers. Due to the obvious importance of the Th1 T cell-rich myocarditis in the pathogenesis of CCC, the focus has been on genes involved in the innate and adaptive immune responses. However, these studies were usually small and led to conflicting results when populations of different ethnicity were studied [10].

As part of the National Heart, Lung and Blood Institute (NHLBI) Retrovirus Epidemiological Donor Study-II (REDS-II), we developed a retrospective cohort study to characterize the natural history of clinical Chagas disease in *T. cruzi* seropositive blood donors^3^. We performed a GWAS study using this well established cohort. All phenotype and genotype data is available upon request.

## Methods

This study is approved by the UCSF CHR, Comissão de Ética para Análise de Projetos de Pesquisa (CAPPesq), Comitê de Ética em Pesquisa da Fundação Hemominas (CEP Hemominas) and National IRB - Brasília: A Comissão Nacional de Ética em Pesquisa (CONEP). Written consent was given by the patients for their information to be stored in the hospital database and used for research.

### Study Design

This study population was derived from a retrospective cohort study, in which 499 *T. cruzi* seropositive (SP) blood donors (cases) identified by blood bank screening (255 from the city of São Paulo and 244 from the city of Montes Claros in the State of Minas Gerais) and 488 seronegative (SN) donors matched by site, donation date (year), age and gender were enrolled^3^. This cohort was supplemented with a total of 101 previously diagnosed cases of cardiomyopathyfrom the Heart Institute of University of Sao Paulo Medical School. From July 2008 to October 2010, all individuals (blood donors and patients with CCC) were characterized by demographic survey (including questions about skin color classification, which is comparable to a race-ethnicity classification in Brazil) and by a health questionnaire and medical evaluation, including electrocardiogram (ECG), echocardiogram (Echo) and laboratory tests. Results of ECG and Echo were reviewed under code at centralized reading centers [11]. The presence of CCC was determined by an expert panel composed of three Brazilian cardiologists based on the evaluation of clinical, laboratory, EKG and Echo findings, as described elsewhere [3]. In brief, a pre-defined set of abnormalities in the Echo or ECG measurements triggered the panel to review cardiac findings blinded to the subject’s serostatus. Each physician is asked to answer if, based on available data for a triggered case, he would diagnose CCC; discordances among the examiners were resolved by consensus. Classification rules were used as general guidance for the diagnosis, as well as the physicians’ experience and clinical expertise. The diagnostic accuracy of this algorithm was very good, with a sensitivity of 98% for the detection of previously diagnosed CCC and specificity of 95%, considering 5% of “false positive” diagnosis of CCC in *T. cruzi* seronegatives donors [3]. In addition to Cardiomyopathy as the main trait, we also evaluated a limited number of specific parameters, including Ejection Fraction (EF), PR interval, QRS duration (QRS), corrected QT interval (QTc), EIA signal/cutoff levels, and *T. cruzi* PCR status [12], for genome-wide association. Of the 600 *T. cruzi* seropositive donors/cases, 221 were classified as having CCC; 311 had no cardiomyopathy and 68 were inconclusive. All samples were submitted for DNA extraction.

### DNA Sample Preparation

DNA was obtained using QIAamp DNA Blood Mini Kit (Qiagen, Hilden, Germany), and quantified using NanoDrop (NanoDrop Technologies, Inc.,Wilmington, DE USA). The concentration accepted was between 50 ng/uL and 100 ng/uL.

### Genotyping

The 600 DNA samples were sent to the Genomics Core Facility at UCSF for genotyping using the Affymetrix Axiom Genome-Wide Latino (Axiom GW LAT 1) array (Affymetrix, Santa Clara, CA), which consists of 818,154 SNPs and is optimized for individuals with ancestry from Europe, West Africa, and Native Americans.

### Genotype Calling, Sample and SNP Filtering

We performed genotype calling using Affymetrix Power Tools (APT) genotyping module. We followed the manufacturer’s genotyping analysis guidelines to remove samples with DishQC below 0.82. QC genotype filtering was performed on a set of SNPs whose genotypes are representative of the expected performance of the standard tiling design, samples with call rate <0.97 were identified and removed. For plate-level QC, all plates passed with plate pass rates >95% and the average call rate of passing samples >99%. Final genotype calling was performed on samples across all SNPs using generic priors. Additional problematic sample were removed from further analysis: samples with Identify By Descent (IBD) score >0.5 and, samples with unknown or contradicted computed gender compared to documented gender. A total of 20 (3%) samples were excluded using these filters.

A series of SNP filtering procedures were performed on the remaining 580 samples. To begin with, the total number of SNPs was 818,154 on array. The filters and their thresholds were: SNP call rate >97%; FLD >3.6; HetSO>3.6; HomRO 2-cluster >0.3; HomRO>3-cluster −0.9; Hardy-Weinberg test P-value >10^−9^; and MAF >0.5%. In all, 142,436 SNPs were removed. The final genotype data set contained 580 samples across 675,718 SNPs that passed all the above filters.

### Genotype Imputation

Genotype imputation was performed using Impute2 version 2.3.0 with 1000 Genomes Phase I integrated variant set as reference panel [13], [14]. A total of 5,767,018 SNPs (including the typed SNPs) were imputed into the Chagas dataset. Imputed genotypes with probability <0.8, SNP call rate <0.7, Hardy-Weinberg test P-value >10^−9^; and MAF >0.5% were removed. The final imputed dataset consisted of 5,486,770 genotypes across the 580 samples.

### Genotype Multidimensional Scaling (MDS)

Assessment for possible population stratification was conducted using Plink-MDS [15]. The Chagas samples were combined with Hapmap populations and MDS was performed on the combined genotypes. The first 4 dimensions were kept as covariates in the subsequent GWAS.

### Phenotyping and GWAS

Genome wide association tests were performed on seven phenotypes: Cardiomyopathy (as defined by the expert panel) [3], PCR, anti- *T cruzi* antibody level (as defined by S/CO value obtained using Ortho *T. cruzi* EIA test system (Raritan,NJ)) [12], ECG measurements (PR, QRS, corrected QT intervals) and ejection fraction (EF) as defined by echocardiogram. For cardiomyopathy, we excluded individuals with inconclusive results determined by the expert panel (total of 67), and analyzed 207 CCC and 306 non-CCC samples. GWAS was performed using snptest_v2.4.1 with the expected genotype counts method (genotype dosages) [13], adjusting for population stratification using the first four dimensions generated in MDS.

### Ancestry Proportion Estimation

**Figure 1:**
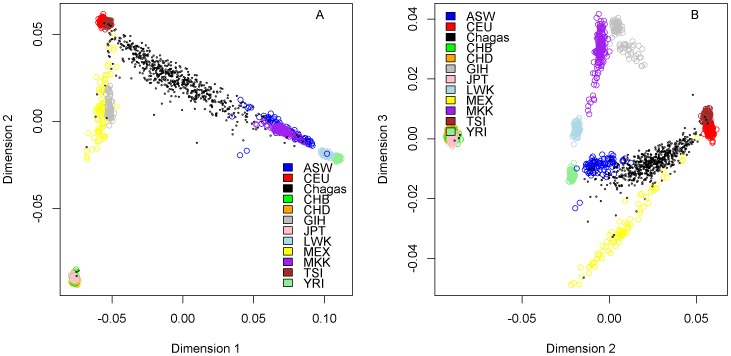
Population stratification of Brazilian Chagas cohort along with Hapmap populations. Each point on the plot represents an individual; each population is coded in a different color. The populations are: ASW: African ancestry in Southwest USA; CEU: Utah residents with Northern and Western European ancestry from the CEPH collection; CHB: Han Chinese in Beijing, China; CHD: Chinese in Metropolitan Denver, Colorado; GIH: Gujarati Indians in Houston, Texas; JPT: Japanese in Tokyo, Japan; LWK: Luhya in Webuye, Kenya; MEX: Mexican ancestry in Los Angeles, California; MKK: Maasai in Kinyawa, Kenya; TSI: Toscans in Italy; YRI: Yoruba in Ibadan, Nigeria.A) Dimension 1 vs. Dimension 2; B) Dimension 2 vs Dimension 3.

Individual ancestry and admixture proportions were estimated using Frappe [16]. Based on visually inspecting Chagas admixture proportions, along with Hapmap populations (Figure 1), four ancestral populations (K = 4) (ASW: African ancestry in Southwest USA; CEU: Utah residents with Northern and Western European ancestry from the CEPH collection; CHB: Han Chinese in Beijing, China; and MEX: Mexican ancestry in Los Angeles, California) were identified and then combined with Chagas genotype data for a total of 239,530 common SNPs. We randomly chose 5% of SNPs for 1000 steps of expectation maximization (EM) optimization to obtain the ancestry proportion estimates for all individuals.

## Results and Discussion

We obtained genome-wide genotypes for 580 Chagas seropositive donors and cases. More than 675,000 SNPs were directly genotyped,and more than 5 million additional genotypes were obtained through genotype imputation. The missing genotype frequency was less than 1% in the final dataset. The average pair-wise concordance of the single control sample across 7 plates was greater than 99.6%.

**Figure 2:**
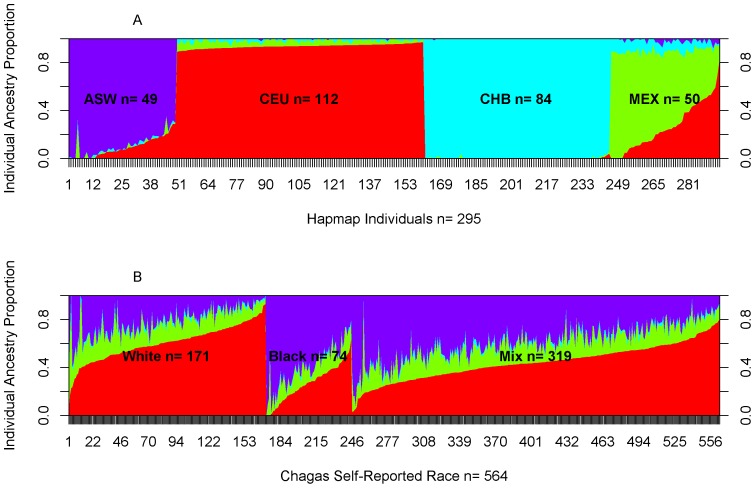
Individual ancestry proportion estimation assuming 4 (K = 4) ancestral populations (color-coded). X-axis shows individuals and y-axis shows ancestry proportions. A) 4 related Hapmap populations: CEU, CHB, ASW, and MEX; B) Chagas cohorts stratified by self-reported races.

The Brazilian population represented in the Chagas cohort displayed a wide range of genetic diversity. Figure 1 shows that the majority of individuals spanned the complete range of admixture proportions between European (CEU and TSI) and African (ASW) populations, with a few individuals of apparent East Asian ancestry (CHB, JPT), and a few individuals showing similarity to Mexican populations (MEX), presumably due mostly to Native American ancestry. Ancestry analysis further quantified the ancestry proportions for each individual in the Chagas cohort (Figure 2). Although 176 individuals self-reported as white race, the majority of them show a high degree of multi-racial ancestry, the same was observed for the self-reported black individuals. Therefore self-reported race is a highly subjective and individual cultural decision, with individuals identifying with both major races displaying a wide range of admixture proportions, which is consistent with previous reports on Brazilian populations [17], [18]. Based on the median of chi-square values resulting from logistic regression in PLINK [15], the genomic inflation factor is 1.086 without adjustment for population stratification. The genomic inflation factor is 1.019 after adjusting for population stratification using the first 4 dimensions generated in MDS.

We detected two SNPs (rs4149018 and rs12582717) which were associated with cardiomyopathy at P<10^−6^. The SNP rs12582717 is located in an intron of the SLCO1B1 gene, while rs4149018 is located in its 5′UTR. SLCO1B1 is a membrane transporter that belongs to a solute carrier family and plays a role in drug metabolism. It is expressed in the liver, brain, heart and kidney, and transports organic anions, such as digoxin, bilirubin, methothrexate and statins. In addition, loss-of function mutations may be associated with impaired drug action in target tissues [19]. Previous studies reported that common variants in SLCO1B1 are strongly associated with an increased risk of statin-induced myopathy [20], [21]. This is the first report of its association with CCC, and while it fails to reach genome-wide significance, the previous associations with a form of myopathy increases the likelihood of its involvement with CCC.

We found a cluster of 12 SNPs within introns of COL14A1 that is associated with PCR positivity. COL14A1is a fibril-associated collagen which interacts with the fibril surface and regulates fibrillogenesis [22]. A previous study showed that COL14A1 harbors a SNP that is associated with HIV-1 viral load during the asymptomatic set-point period of infection [23].

All SNPs that are significantly associated with any of the seven traits can be found in Table S1. Table 1 lists a total of 46 genic SNPs that are associated with the seven traits with P<10^−6^. None of the traits are associated with SNPs with P<10^−7^, except PR interval, which shows association with six SNPs at P<10^−7^. The six SNPs are located in six different genes: ABCB5, COL1A2, HSPB8, ACCN1, LEPREL4, and LPIN2.

**Table 1:** Genomic positions that associated to traits (unadjusted P-value <10^−6^, intergenic positions not shown).

Trait	Chro	Rsid	Pos	P-value	Gene:Annotation	Gene Information
Cardio	12p12.2	rs4149018	21291561	1.2×10**^−^** ^7^	SLCO1B1∶5utr	solute carrier organic anion transporter family, member 1B1
Cardio	12p12.2	rs12582717	21296806	2.3×10**^−^** ^7^	SLCO1B1:intron	solute carrier organic anion transporter family, member 1B1
EF	8p23.1	rs2645430	11659109	3.7×10**^−^** ^7^	FDFT1:upstream	farnesyl-diphosphatefarnesyltransferase 1
EF	8p23.1	rs1497042	11660614	4.5×10**^−^** ^7^	FDFT1:intron	farnesyl-diphosphatefarnesyltransferase 1
EF	8p23.1	rs2252567	11660916	5.7×10**^−^** ^7^	FDFT1:intron	farnesyl-diphosphatefarnesyltransferase 1
EF	10q24.31	rs111981433	102733776	7.9×10**^−^** ^7^	MRPL43:downstream	mitochondrial ribosomal protein L43
EF	10q24.31	rs807029	102764047	4.4×10**^−^** ^7^	LZTS2:intron	leucine zipper, putative tumor suppressor 2
EIA	3p14.1	rs13078828	66479487	4.0×10**^−^** ^7^	LRIG1:intron	leucine-rich repeats and immunoglobulin-like domains 1
PCR	8p21.3	rs57302454	22938661	5.7×10**^−^** ^7^	LOC254896:upstream	–
PCR	8q24.12	rs7013781	121214291	9.0×10**^−^** ^7^	COL14A1:intron	collagen, type XIV, alpha 1
PCR	8q24.12	rs2054148	121216510	8.6×10**^−^** ^7^	COL14A1:intron	collagen, type XIV, alpha 1
PCR	8q24.12	rs10955961	121218601	2.8×10**^−^** ^7^	COL14A1:intron	collagen, type XIV, alpha 1
PCR	8q24.12	rs10955962	121219597	5.0×10**^−^** ^7^	COL14A1:intron	collagen, type XIV, alpha 1
PCR	8q24.12	rs2034842	121224037	6.4×10**^−^** ^7^	COL14A1:intron	collagen, type XIV, alpha 1
PCR	8q24.12	rs10808508	121227068	4.2×10**^−^** ^7^	COL14A1:intron	collagen, type XIV, alpha 1
PCR	8q24.12	rs7387094	121230787	4.9×10**^−^** ^7^	COL14A1:intron	collagen, type XIV, alpha 1
PCR	8q24.12	rs7387373	121230950	8.4×10**^−^** ^7^	COL14A1:intron	collagen, type XIV, alpha 1
PCR	8q24.12	rs56159702	121231250	3.8×10**^−^** ^7^	COL14A1:intron	collagen, type XIV, alpha 1
PCR	8q24.12	rs58829748	121231299	7.2×10**^−^** ^7^	COL14A1:intron	collagen, type XIV, alpha 1
PCR	8q24.12	rs6989074	121234756	5.0×10**^−^** ^7^	COL14A1:intron	collagen, type XIV, alpha 1
PCR	11q24.2	rs4408325	124821575	1.4×10**^−^** ^7^	CCDC15:upstream	coiled-coil domain containing 15
PCR	18q12.2	rs116303449	32956931	5.9×10**^−^** ^7^	ZNF396∶5utr	zinc finger protein 396
PR	2p13.2	rs56988800	71688118	1.3×10**^−^** ^7^	DYSF:intron	dysferlin, limb girdle muscular dystrophy 2B (autosomal recessive)
PR	2p13.2	rs79523511	71692883	1.8×10^−7^	DYSF:intron	dysferlin, limb girdle muscular dystrophy 2B(autosomal recessive)
PR	3p26.3	rs76090503	1129819	4.0×10^−7^	CNTN6:upstream	contactin 6
PR	3p26.3	rs9815195	1301130	7.4×10^−7^	CNTN6:intron	contactin 6
PR	4p15.32	rs114133078	15354385	2.2×10^−7^	C1QTNF7:intron	C1q and tumor necrosis factor related protein 7
PR	6p21.1	rs61018535	43449230	1.5×10^−7^	TJAP1∶5utr	tight junction associated protein 1 (peripheral)
PR	7p21.1	rs59623110	20678749	2.7×10^−7^	ABCB5:intron	ATP-binding cassette, sub-family B (MDR/TAP), member 5
PR	7p21.1	rs73276602	20681850	9.9×10^−8^	ABCB5:intron	ATP-binding cassette, sub-family B (MDR/TAP), member 5
PR	7q21.12	rs114137957	87374869	9.6×10^−7^	RUNDC3B:intron	RUN domain containing 3B
PR	7q21.3	rs115744676	94061404	6.9×10^−9^	COL1A2:downstream	collagen, type I, alpha 2
PR	7q21.3	rs116187617	94061601	1.2×10^−7^	COL1A2:downstream	collagen, type I, alpha 2
PR	8p12	rs75609241	29082285	4.5×10^−7^	KIF13B:intron	kinesin family member 13B
PR	12q24.23	rs78852656	119619997	3.9×10^−8^	HSPB8:intron	heat shock 22 kDa protein 8
PR	17p13.3	rs59403466	2785895	6.4×10^−7^	RAP1GAP2:intron	RAP1 GTPase activating protein 2
PR	17q12	rs117364231	32306423	9.7×10^−8^	ACCN1:intron	Unknown
PR	17q21.1	rs75041531	38393151	9.4×10^−7^	WIPF2∶5utr	WAS/WASL interacting protein family, member 2
PR	17q21.2	rs191392302	39953961	3.9×10^−8^	LEPREL4:downstream	leprecan-like 4
PR	18p11.32	rs11664027	807368	2.5×10^−7^	YES1∶5utr	v-yes-1 Yamaguchi sarcoma viral oncogene homolog 1
PR	18p11.31	rs8087073	2967845	1.1×10^−9^	LPIN2∶5utr	lipin 2
PR	20p11.21	rs3176130	23031656	1.5×10^−7^	THBD:upstream	Thrombomodulin
QRS	6p21.31	rs182503338	34582274	7.9×10^−7^	C6orf106:intron	chromosome 6 open reading frame 106
QRS	10p12.1	rs72786268	25770025	3.8×10^−7^	GPR158:intron	G protein-coupled receptor 158
QRS	11p15.4	rs10769783	7396191	2.1×10^−7^	SYT9:intron	synaptotagmin IX
QRS	14q13.1	rs2274511	33829320	8.4×10^−7^	NPAS3:intron	neuronal PAS domain protein 3

HSPB8/Hsp22/H11 is a small heat shock protein whose heart-specific overexpression induces myocardial hypertrophy [24]. Furthermore, HSPB8-transgenic mice bearing the K141N mutation expressed myocardial hypertrophy, ventricular dysfunction and apical fibrosis-the latter being a hallmark of heart involvement in CCC [25]. Significantly, expression of HSPB8 is selectively increased in myocardial tissue from CCC patients, rather than in idiopathic dilated cardiomyopathy patients [26].

ACCN1/ASIC2 is an acid-sensing ion channel which has been implicated as a mechanoreceptor/baroreceptor in afferent sympathetic nerve fibers. ACCN1/ASIC2 -null mice show an exaggerated sympathetic and depressed parasympathetic control of the circulation, indicative of an impaired baroreceptor reflex. Multiple measures of baroreceptor activity each suggest that mechano-sensitivity is diminished in ASIC2 null mice. This recapitulates the pathological dysautonomia seen in heart failure [27]. Significantly, dysautonomia with reduced baroreflex sensitivity is a hallmark of Chagas disease and progresses with disease severity and may be directly linked to the increased PR interval phenotype [28], [29].

COL1A2 was found to be increased in hearts from idiopathic dilated cardiomyopathy patients [30]. ABCB5 is another membrane transporter that belongs to the ATP-binding cassette (ABC) transporter family, which has been implicated in cancer progression and resistance to chemotherapy [31]. Polymorphisms in the LPIN2 gene are associated with metabolic disease traits, including insulin sensitivity, diabetes, and blood pressure [32]. LEPREL4 is a nucleolar protein that is associated with chromosomes during mitosis [33].

The genes harboring the SNPs found to be associated with diagnosed CCC and cardiac phenotypes in this GWAS showed no overlap with the hypothesis-driven candidate genes studied so far in CCC. Due to the relevance of inflammation and myocarditis in the pathogenesis of CCC, most of the candidate genes and SNPs chosen for study were previously known or suspected to lead to variations in the intensity of the innate or acquired immune response, as well as inflammatory cell migration (reviewed by Teixeira [34]). Among the innate and adaptive immunity candidate genes studied, alleles of HLA class I (HLA-C locus) and class II (DRB1 and DQB1 loci), and SNPs in IL1B, IL1RN, IL10, IL12B, TNFA, LTA, BAT-1/UAP56, NFkBIL-1 (the latter four located in the MHC class III region in chromosome 6), and MAL/TIRAP [35] have been found previously to be associated to CCC. The TNFA −308 promoter polymorphism is associated with survival in CCC patients with ventricular dysfunction [36].

Among genes encoding chemokines and chemokine receptors, polymorphisms associated with CCC have been found, including MCP1/CCL2 [37]. CCR5, MIG/CXCL9, and IP10/CXCL10 (the latter associated with severe CCC, with ventricular dysfuction). Significantly, the CXCL9 polymorphism was associated with increased levels of myocardial CXCL9 expression and myocarditis; conversely, myocardial CXCL9 levels were found to correlate with intensity of myocarditis [38].

In summary, our GWAS results suggest that polymorphisms in the SLCO1B1 gene are associated with the cardiomyopathy phenotype, whereas polymorphisms in heat shock protein HSPB8, the ion channel ACCN1, and alpha-2 type I collagen COL1A2, involved in cardiac hypertrophy, baroreflex sensitivity and autonomic control of the circulation, and fibrosis, respectively, are associated with the PR interval. However, none of the loci detected in this study have been linked to PR interval in previous primary or meta-analysis studies of cardiac disease [39]. This may suggest that the mechanism of CCC may be different from previous studies or our statistical power to detect true association is limited by the moderate sample size and high degree of genetic diversity of our cohort. Together with the available literature, results indicate that both cardiovascular-related and immune-related gene polymorphisms may play a role in the genetic predisposition to CCC development.

## Supporting Information

Table S1 **SNPs that are significantly associated with any of the seven traits with P<10^−5^.**
(DOCX)
