# Supplementary material for: Genome Wide Association Study (GWAS) of Chagas Cardiomyopathy in Trypanosoma cruzi Seropositive Subjects
Source: PLoS One. 2013 Nov 20;8(11):e79629. doi: 10.1371/journal.pone.0079629 (PMC3854669; doi:10.1371/journal.pone.0079629)
Supplement: Table S1 — SNPs that are significantly associated with any of the seven traits with P<10−5. (DOCX) [file pone.0079629.s001.docx]

Supplementary Table S1: SNPs that are significantly associated with any of the seven traits with P<10^-5^

| Phenotype | Chro | rsid | pos | allele_A | allele_B | P-value |
| --- | --- | --- | --- | --- | --- | --- |
| Cardiomyopathy | 1 | rs2480054 | 14792535 | A | G | 3.60E-06 |
| Cardiomyopathy | 2 | chr2:56720215:D | 56720215 | CCT | C | 1.74E-06 |
| Cardiomyopathy | 3 | rs78356356 | 69824093 | A | G | 5.39E-06 |
| Cardiomyopathy | 6 | rs6910233 | 33534726 | G | T | 3.88E-06 |
| Cardiomyopathy | 6 | rs182503338 | 34582274 | T | G | 1.45E-06 |
| Cardiomyopathy | 6 | rs2038868 | 40328378 | A | G | 9.03E-06 |
| Cardiomyopathy | 6 | rs35131064 | 44172063 | C | T | 6.25E-06 |
| Cardiomyopathy | 8 | rs2517175 | 17459430 | A | T | 3.94E-06 |
| Cardiomyopathy | 8 | rs2588131 | 17460753 | C | T | 3.90E-06 |
| Cardiomyopathy | 8 | rs7829987 | 17462222 | T | G | 3.50E-06 |
| Cardiomyopathy | 8 | rs12542743 | 32318355 | T | C | 3.60E-06 |
| Cardiomyopathy | 8 | rs10954846 | 32320220 | G | A | 5.48E-06 |
| Cardiomyopathy | 9 | chr9:113590717:I | 1.14E+08 | C | CT | 5.14E-06 |
| Cardiomyopathy | 10 | chr10:14886486:D | 14886486 | CG | C | 7.20E-06 |
| Cardiomyopathy | 11 | rs10769783 | 7396191 | A | G | 6.21E-06 |
| Cardiomyopathy | 12 | rs7964992 | 19016296 | T | A | 7.41E-06 |
| Cardiomyopathy | 12 | rs10437758 | 19018632 | C | T | 7.47E-06 |
| Cardiomyopathy | 12 | rs1490734 | 19019467 | T | A | 5.79E-06 |
| Cardiomyopathy | 12 | rs12319113 | 19024558 | G | A | 1.25E-06 |
| Cardiomyopathy | 12 | rs4149018 | 21291561 | T | G | 1.19E-07 |
| Cardiomyopathy | 12 | rs12582717 | 21296806 | C | G | 2.31E-07 |
| Cardiomyopathy | 14 | chr14:22193551:D | 22193551 | GC | G | 4.52E-06 |
| Cardiomyopathy | 14 | chr14:22193552:D | 22193552 | CA | C | 2.54E-06 |
| Cardiomyopathy | 14 | rs11157162 | 22194319 | T | C | 6.30E-06 |
| Cardiomyopathy | 14 | rs8022088 | 22195531 | G | A | 7.27E-06 |
| Cardiomyopathy | 14 | chr14:22195687:I | 22195687 | C | CT | 5.56E-06 |
| Cardiomyopathy | 14 | rs10132760 | 22196541 | A | C | 5.36E-06 |
| Cardiomyopathy | 14 | rs1997532 | 22197477 | T | C | 9.26E-06 |
| Cardiomyopathy | 14 | rs8011509 | 22201104 | T | C | 8.51E-06 |
| Cardiomyopathy | 14 | rs8006524 | 22201150 | C | T | 9.20E-06 |
| Cardiomyopathy | 14 | rs7150049 | 22201888 | A | G | 7.77E-06 |
| Cardiomyopathy | 14 | rs8011979 | 22201900 | C | T | 9.09E-06 |
| Cardiomyopathy | 14 | rs11157167 | 22202711 | T | C | 5.58E-06 |
| Cardiomyopathy | 14 | rs35955841 | 65319361 | G | A | 3.04E-06 |
| Cardiomyopathy | 15 | rs10468092 | 38434891 | A | G | 4.17E-06 |
| Cardiomyopathy | 15 | rs1356410 | 42434837 | T | C | 7.82E-06 |
| Cardiomyopathy | 19 | rs2262909 | 22135270 | A | C | 3.91E-06 |
| Cardiomyopathy | 21 | rs73199525 | 32884848 | G | A | 7.25E-06 |
| Cardiomyopathy | 22 | rs5760472 | 24953571 | A | G | 9.83E-06 |
| Cardiomyopathy | 22 | rs4493363 | 35144411 | G | A | 4.41E-06 |
| Phenotype | Chromosome | rsid | pos | allele_A | allele_B | EF_50_frequentist_add_expected_pvalue |
| EjectionFraction | 1 | rs2235701 | 40883095 | C | T | 7.07E-06 |
| EjectionFraction | 1 | rs74133262 | 1.86E+08 | C | T | 9.96E-07 |
| EjectionFraction | 4 | rs72927357 | 1.02E+08 | A | C | 9.07E-06 |
| EjectionFraction | 5 | rs56160829 | 57460834 | G | T | 1.29E-06 |
| EjectionFraction | 5 | rs2962010 | 57473703 | G | C | 2.12E-06 |
| EjectionFraction | 5 | rs2964182 | 57476508 | G | T | 2.27E-06 |
| EjectionFraction | 5 | rs2964183 | 57477809 | G | C | 1.43E-06 |
| EjectionFraction | 5 | rs2964186 | 57480693 | C | T | 8.04E-07 |
| EjectionFraction | 5 | rs73757719 | 57487665 | T | C | 3.07E-06 |
| EjectionFraction | 5 | rs2409014 | 57494453 | T | A | 3.29E-06 |
| EjectionFraction | 5 | rs7713811 | 57496382 | T | G | 1.03E-06 |
| EjectionFraction | 5 | rs112730887 | 62240926 | T | C | 4.95E-06 |
| EjectionFraction | 5 | rs6879663 | 1.69E+08 | A | C | 9.63E-06 |
| EjectionFraction | 6 | rs73725220 | 16700060 | G | A | 3.13E-06 |
| EjectionFraction | 6 | chr6:88889267:I | 88889267 | C | CG | 5.90E-06 |
| EjectionFraction | 6 | rs17781283 | 1.38E+08 | G | A | 1.94E-06 |
| EjectionFraction | 7 | rs60111824 | 6774135 | A | G | 8.78E-06 |
| EjectionFraction | 7 | rs35205176 | 51615152 | T | A | 1.74E-06 |
| EjectionFraction | 7 | rs4557653 | 51615505 | G | A | 4.38E-06 |
| EjectionFraction | 7 | rs11769683 | 51616830 | G | A | 6.14E-06 |
| EjectionFraction | 7 | rs13239514 | 51624353 | C | T | 3.71E-06 |
| EjectionFraction | 7 | rs12538073 | 51625595 | T | C | 4.15E-06 |
| EjectionFraction | 7 | rs4439078 | 51626326 | T | C | 4.42E-06 |
| EjectionFraction | 7 | rs13230827 | 51629529 | G | A | 4.19E-06 |
| EjectionFraction | 7 | rs17636421 | 51633059 | T | C | 4.34E-06 |
| EjectionFraction | 7 | chr7:51634762:I | 51634762 | C | CTG | 3.73E-06 |
| EjectionFraction | 7 | rs7798734 | 90928757 | G | T | 3.62E-06 |
| EjectionFraction | 7 | rs7802175 | 90928923 | A | G | 4.16E-06 |
| EjectionFraction | 7 | chr7:90937147:I | 90937147 | G | GA | 4.86E-06 |
| EjectionFraction | 7 | rs55646004 | 90940897 | C | T | 2.30E-06 |
| EjectionFraction | 7 | rs747236 | 90943564 | C | T | 2.27E-06 |
| EjectionFraction | 7 | rs1429524 | 90944268 | C | G | 1.74E-06 |
| EjectionFraction | 7 | rs7777183 | 1.22E+08 | C | A | 1.60E-06 |
| EjectionFraction | 7 | rs2080045 | 1.22E+08 | A | G | 1.37E-06 |
| EjectionFraction | 7 | rs1860859 | 1.22E+08 | T | C | 1.66E-06 |
| EjectionFraction | 7 | rs6466831 | 1.22E+08 | G | A | 1.78E-06 |
| EjectionFraction | 7 | rs6466832 | 1.22E+08 | T | C | 1.31E-06 |
| EjectionFraction | 7 | rs2109965 | 1.22E+08 | C | T | 6.22E-06 |
| EjectionFraction | 7 | rs9770057 | 1.22E+08 | G | C | 5.64E-06 |
| EjectionFraction | 7 | rs6466839 | 1.22E+08 | C | T | 6.12E-06 |
| EjectionFraction | 7 | rs2402620 | 1.22E+08 | T | C | 4.91E-06 |
| EjectionFraction | 7 | rs7794272 | 1.22E+08 | A | G | 2.48E-06 |
| EjectionFraction | 7 | rs6948815 | 1.22E+08 | T | G | 5.28E-06 |
| EjectionFraction | 7 | rs11982969 | 1.42E+08 | G | A | 3.62E-06 |
| EjectionFraction | 8 | rs4841579 | 11516592 | T | G | 1.45E-06 |
| EjectionFraction | 8 | rs13262332 | 11551982 | G | A | 5.72E-06 |
| EjectionFraction | 8 | rs2645430 | 11659109 | A | G | 3.68E-07 |
| EjectionFraction | 8 | rs1497042 | 11660614 | C | T | 4.53E-07 |
| EjectionFraction | 8 | rs2252567 | 11660916 | C | T | 5.67E-07 |
| EjectionFraction | 8 | chr8:11664532:D | 11664532 | TACAAATAAAA | T | 2.40E-06 |
| EjectionFraction | 8 | chr8:11664606:D | 11664606 | CTA | C | 7.60E-06 |
| EjectionFraction | 8 | chr8:11665095:D | 11665095 | TA | T | 3.83E-06 |
| EjectionFraction | 8 | rs1736057 | 11665217 | G | A | 4.01E-06 |
| EjectionFraction | 8 | rs2285232 | 91546162 | C | G | 5.34E-06 |
| EjectionFraction | 8 | rs1858673 | 91551526 | C | A | 5.56E-06 |
| EjectionFraction | 8 | chr8:91551546:I | 91551546 | A | ACT | 5.56E-06 |
| EjectionFraction | 8 | rs756568 | 91557038 | G | C | 6.20E-06 |
| EjectionFraction | 8 | rs10086462 | 91558089 | A | G | 6.37E-06 |
| EjectionFraction | 8 | rs10091275 | 91559751 | A | G | 9.42E-06 |
| EjectionFraction | 8 | rs7840846 | 91565501 | T | A | 6.11E-06 |
| EjectionFraction | 8 | rs7819519 | 91565504 | C | T | 2.55E-06 |
| EjectionFraction | 8 | rs4735224 | 91567046 | C | A | 2.53E-06 |
| EjectionFraction | 8 | rs7833025 | 91568470 | G | C | 5.95E-06 |
| EjectionFraction | 8 | rs4734246 | 91569028 | A | C | 3.98E-06 |
| EjectionFraction | 8 | rs4735225 | 91569100 | G | C | 6.51E-06 |
| EjectionFraction | 8 | rs4734247 | 91569144 | A | G | 2.52E-06 |
| EjectionFraction | 8 | rs4735226 | 91569238 | T | A | 4.42E-06 |
| EjectionFraction | 8 | rs10808615 | 91569704 | A | T | 6.11E-06 |
| EjectionFraction | 8 | rs1018836 | 91570362 | A | G | 2.45E-06 |
| EjectionFraction | 8 | rs4735230 | 91574184 | G | C | 6.15E-06 |
| EjectionFraction | 8 | rs10956720 | 91576222 | C | T | 5.55E-06 |
| EjectionFraction | 8 | rs7823461 | 91581075 | T | C | 5.29E-06 |
| EjectionFraction | 8 | rs5005585 | 91581975 | A | G | 2.50E-06 |
| EjectionFraction | 9 | rs181881383 | 25192573 | C | T | 2.23E-08 |
| EjectionFraction | 10 | rs34174118 | 1452537 | G | A | 2.67E-06 |
| EjectionFraction | 10 | rs17293817 | 1452786 | G | A | 2.46E-06 |
| EjectionFraction | 10 | rs34510805 | 1453878 | G | A | 5.36E-06 |
| EjectionFraction | 10 | rs59132240 | 25591153 | A | G | 5.55E-06 |
| EjectionFraction | 10 | rs55801305 | 25591409 | T | C | 6.90E-06 |
| EjectionFraction | 10 | chr10:25605832:D | 25605832 | GTGTGTGTT | G | 9.30E-06 |
| EjectionFraction | 10 | rs57214420 | 1.03E+08 | G | A | 5.29E-06 |
| EjectionFraction | 10 | rs56317403 | 1.03E+08 | T | G | 6.29E-06 |
| EjectionFraction | 10 | rs3802724 | 1.03E+08 | T | C | 6.31E-06 |
| EjectionFraction | 10 | rs4917916 | 1.03E+08 | G | A | 6.31E-06 |
| EjectionFraction | 10 | chr10:102679269:D | 1.03E+08 | GA | G | 5.00E-06 |
| EjectionFraction | 10 | rs12241232 | 1.03E+08 | T | C | 2.55E-06 |
| EjectionFraction | 10 | rs55942016 | 1.03E+08 | G | A | 2.55E-06 |
| EjectionFraction | 10 | rs7911957 | 1.03E+08 | A | G | 2.54E-06 |
| EjectionFraction | 10 | rs2863094 | 1.03E+08 | C | T | 2.13E-06 |
| EjectionFraction | 10 | chr10:102700479:D | 1.03E+08 | TCCTTCCCTC | T | 1.31E-06 |
| EjectionFraction | 10 | rs10883565 | 1.03E+08 | T | C | 1.67E-06 |
| EjectionFraction | 10 | chr10:102705058:I | 1.03E+08 | A | AT | 8.76E-07 |
| EjectionFraction | 10 | rs4919509 | 1.03E+08 | C | T | 4.43E-06 |
| EjectionFraction | 10 | rs1056295 | 1.03E+08 | T | C | 1.69E-06 |
| EjectionFraction | 10 | rs11190773 | 1.03E+08 | G | C | 4.08E-06 |
| EjectionFraction | 10 | rs927302 | 1.03E+08 | G | C | 1.62E-06 |
| EjectionFraction | 10 | rs1570171 | 1.03E+08 | T | A | 1.43E-06 |
| EjectionFraction | 10 | rs41291460 | 1.03E+08 | A | C | 2.81E-06 |
| EjectionFraction | 10 | rs41291462 | 1.03E+08 | C | A | 3.00E-06 |
| EjectionFraction | 10 | rs111981433 | 1.03E+08 | C | T | 7.89E-07 |
| EjectionFraction | 10 | rs12253241 | 1.03E+08 | T | C | 2.42E-06 |
| EjectionFraction | 10 | rs4919510 | 1.03E+08 | C | G | 1.69E-06 |
| EjectionFraction | 10 | rs735137 | 1.03E+08 | C | G | 2.05E-06 |
| EjectionFraction | 10 | rs67813203 | 1.03E+08 | A | G | 2.52E-06 |
| EjectionFraction | 10 | rs67692077 | 1.03E+08 | T | C | 3.20E-06 |
| EjectionFraction | 10 | rs12571302 | 1.03E+08 | C | A | 3.11E-06 |
| EjectionFraction | 10 | rs2295716 | 1.03E+08 | T | C | 2.27E-06 |
| EjectionFraction | 10 | rs2863095 | 1.03E+08 | C | T | 2.98E-06 |
| EjectionFraction | 10 | rs3740485 | 1.03E+08 | C | T | 7.32E-06 |
| EjectionFraction | 10 | rs3740486 | 1.03E+08 | T | C | 7.32E-06 |
| EjectionFraction | 10 | rs3740487 | 1.03E+08 | C | A | 7.37E-06 |
| EjectionFraction | 10 | rs3824783 | 1.03E+08 | G | A | 7.47E-06 |
| EjectionFraction | 10 | rs112309064 | 1.03E+08 | G | A | 2.07E-06 |
| EjectionFraction | 10 | rs111545834 | 1.03E+08 | C | T | 9.30E-06 |
| EjectionFraction | 10 | rs4919511 | 1.03E+08 | G | A | 2.73E-06 |
| EjectionFraction | 10 | rs3740488 | 1.03E+08 | A | T | 8.25E-06 |
| EjectionFraction | 10 | rs3740489 | 1.03E+08 | A | G | 5.83E-06 |
| EjectionFraction | 10 | rs3740490 | 1.03E+08 | G | C | 3.61E-06 |
| EjectionFraction | 10 | rs11190786 | 1.03E+08 | A | G | 2.17E-06 |
| EjectionFraction | 10 | rs7902510 | 1.03E+08 | C | T | 3.30E-06 |
| EjectionFraction | 10 | rs7903239 | 1.03E+08 | G | T | 3.42E-06 |
| EjectionFraction | 10 | rs55646950 | 1.03E+08 | C | T | 3.16E-06 |
| EjectionFraction | 10 | rs750866 | 1.03E+08 | A | G | 3.03E-06 |
| EjectionFraction | 10 | rs701834 | 1.03E+08 | C | T | 3.01E-06 |
| EjectionFraction | 10 | rs701835 | 1.03E+08 | A | G | 3.05E-06 |
| EjectionFraction | 10 | rs807029 | 1.03E+08 | C | T | 4.41E-07 |
| EjectionFraction | 10 | rs807027 | 1.03E+08 | T | A | 3.50E-06 |
| EjectionFraction | 10 | rs807025 | 1.03E+08 | C | T | 8.59E-06 |
| EjectionFraction | 11 | rs10895846 | 1.06E+08 | A | C | 3.58E-06 |
| EjectionFraction | 11 | rs6591137 | 1.06E+08 | C | T | 5.95E-06 |
| EjectionFraction | 11 | rs11226814 | 1.06E+08 | T | C | 3.12E-06 |
| EjectionFraction | 11 | chr11:105507922:D | 1.06E+08 | GC | G | 3.07E-06 |
| EjectionFraction | 11 | rs56034429 | 1.06E+08 | T | G | 3.12E-06 |
| EjectionFraction | 11 | chr11:105508190:I | 1.06E+08 | A | AT | 3.04E-06 |
| EjectionFraction | 11 | rs10895847 | 1.06E+08 | A | G | 7.29E-06 |
| EjectionFraction | 11 | rs4261254 | 1.06E+08 | T | C | 3.80E-06 |
| EjectionFraction | 11 | rs10895849 | 1.06E+08 | C | T | 3.87E-06 |
| EjectionFraction | 11 | rs2409574 | 1.06E+08 | C | G | 4.71E-06 |
| EjectionFraction | 11 | rs4301761 | 1.06E+08 | G | C | 7.05E-06 |
| EjectionFraction | 11 | rs4754134 | 1.06E+08 | C | T | 5.58E-06 |
| EjectionFraction | 11 | rs3170 | 1.06E+08 | A | T | 2.68E-06 |
| EjectionFraction | 13 | rs9525562 | 42611667 | C | A | 8.84E-06 |
| EjectionFraction | 14 | rs6572843 | 52864237 | C | T | 4.40E-06 |
| EjectionFraction | 16 | rs12926788 | 24879963 | C | G | 4.50E-06 |
| EjectionFraction | 18 | rs3786431 | 3814148 | C | G | 1.94E-06 |
| EjectionFraction | 19 | rs6510683 | 2547015 | A | C | 5.49E-06 |
| EjectionFraction | 19 | rs185543003 | 2714170 | T | C | 3.66E-06 |
| Phenotype | Chromosome | rsid | pos | allele_A | allele_B | EIA_frequentist_add_expected_pvalue |
| EIA | 2 | rs13399257 | 1.22E+08 | A | G | 9.73E-06 |
| EIA | 3 | rs13078828 | 66479487 | G | A | 4.00E-07 |
| EIA | 3 | rs114774257 | 1.46E+08 | T | C | 4.88E-06 |
| EIA | 4 | rs4974669 | 2286850 | C | G | 8.57E-06 |
| EIA | 4 | chr4:2286908:I | 2286908 | A | AG | 9.13E-06 |
| EIA | 4 | rs11722299 | 2288238 | A | G | 8.73E-06 |
| EIA | 4 | rs11723429 | 2288626 | T | G | 4.12E-06 |
| EIA | 4 | rs4602560 | 2292921 | A | G | 1.85E-06 |
| EIA | 5 | rs72792924 | 1.25E+08 | T | A | 5.20E-06 |
| EIA | 5 | rs76815036 | 1.25E+08 | C | T | 3.13E-06 |
| EIA | 5 | rs17153722 | 1.25E+08 | C | T | 3.13E-06 |
| EIA | 5 | rs11241851 | 1.25E+08 | A | G | 3.44E-06 |
| EIA | 5 | rs11241852 | 1.25E+08 | G | T | 2.95E-06 |
| EIA | 5 | rs11241853 | 1.25E+08 | A | C | 2.87E-06 |
| EIA | 5 | rs12152825 | 1.25E+08 | G | A | 2.87E-06 |
| EIA | 5 | rs59002887 | 1.25E+08 | A | G | 2.86E-06 |
| EIA | 5 | rs59562349 | 1.25E+08 | C | A | 2.85E-06 |
| EIA | 5 | rs17153728 | 1.25E+08 | A | G | 2.83E-06 |
| EIA | 5 | rs72795058 | 1.25E+08 | G | C | 2.80E-06 |
| EIA | 5 | rs72795060 | 1.25E+08 | A | G | 2.82E-06 |
| EIA | 5 | rs55673267 | 1.25E+08 | T | C | 2.81E-06 |
| EIA | 5 | rs2221261 | 1.25E+08 | A | C | 2.79E-06 |
| EIA | 5 | rs1910003 | 1.25E+08 | G | A | 2.43E-06 |
| EIA | 5 | rs59663074 | 1.25E+08 | A | G | 2.78E-06 |
| EIA | 5 | rs58014928 | 1.25E+08 | G | A | 2.79E-06 |
| EIA | 5 | rs72795063 | 1.25E+08 | T | A | 2.78E-06 |
| EIA | 5 | rs72795065 | 1.25E+08 | T | A | 2.78E-06 |
| EIA | 5 | rs72795066 | 1.25E+08 | A | G | 2.78E-06 |
| EIA | 5 | rs72795068 | 1.25E+08 | G | A | 2.78E-06 |
| EIA | 5 | rs11241854 | 1.25E+08 | A | T | 2.96E-06 |
| EIA | 5 | rs12374456 | 1.25E+08 | C | T | 2.92E-06 |
| EIA | 5 | rs12374458 | 1.25E+08 | G | C | 2.91E-06 |
| EIA | 5 | rs34705560 | 1.25E+08 | G | A | 2.76E-06 |
| EIA | 5 | rs4082667 | 1.25E+08 | G | C | 2.75E-06 |
| EIA | 6 | rs6934608 | 6169890 | T | G | 1.17E-06 |
| EIA | 6 | rs4706434 | 71060696 | A | G | 9.56E-06 |
| EIA | 9 | rs78925264 | 9015087 | T | C | 9.22E-06 |
| EIA | 10 | rs6481260 | 58963458 | T | C | 5.73E-06 |
| EIA | 10 | chr10:58965972:I | 58965972 | C | CAT | 5.68E-06 |
| EIA | 10 | rs7083404 | 58972355 | T | A | 5.77E-06 |
| EIA | 10 | rs11005694 | 58974808 | C | G | 5.58E-06 |
| EIA | 10 | rs7908694 | 58997325 | T | C | 9.73E-06 |
| EIA | 10 | rs10826010 | 59004770 | G | T | 9.59E-06 |
| EIA | 11 | rs17110679 | 1.1E+08 | T | A | 8.39E-06 |
| EIA | 12 | chr12:15193677:D | 15193677 | GAA | G | 1.53E-07 |
| EIA | 12 | chr12:64724815:I | 64724815 | T | TA | 8.73E-06 |
| EIA | 12 | rs7487873 | 64727456 | A | C | 4.87E-06 |
| EIA | 12 | rs10878150 | 64728921 | G | C | 9.77E-06 |
| EIA | 12 | rs6581543 | 64729745 | A | G | 8.60E-06 |
| EIA | 12 | rs12146838 | 92375595 | A | T | 3.76E-06 |
| EIA | 12 | rs11106382 | 92377981 | C | G | 7.96E-06 |
| EIA | 13 | rs12430077 | 52609208 | T | C | 4.52E-06 |
| EIA | 13 | rs186967887 | 71249529 | T | A | 5.64E-06 |
| EIA | 14 | rs2283391 | 72802433 | T | C | 1.39E-06 |
| EIA | 14 | rs1520332 | 72802666 | G | A | 4.46E-06 |
| EIA | 14 | rs1520331 | 72802736 | T | C | 4.89E-06 |
| EIA | 14 | rs2283390 | 72803482 | C | A | 4.73E-06 |
| EIA | 14 | rs1915132 | 72805490 | T | C | 1.92E-06 |
| EIA | 14 | rs2681771 | 72806117 | C | A | 2.18E-06 |
| EIA | 14 | rs2239243 | 72806264 | G | A | 2.66E-06 |
| EIA | 14 | rs2239241 | 72807042 | G | A | 1.93E-06 |
| EIA | 14 | rs2681769 | 72807278 | T | C | 2.00E-06 |
| EIA | 14 | rs2681768 | 72807405 | C | T | 2.00E-06 |
| EIA | 14 | rs2079506 | 72807559 | A | G | 1.67E-06 |
| EIA | 14 | rs2681767 | 72807622 | G | A | 1.64E-06 |
| EIA | 14 | rs2079505 | 72807663 | A | G | 2.00E-06 |
| EIA | 14 | rs2079504 | 72807682 | C | T | 1.95E-06 |
| EIA | 14 | rs8019358 | 72807815 | T | A | 2.00E-06 |
| EIA | 14 | chr14:72807899:D | 72807899 | CA | C | 2.09E-06 |
| EIA | 14 | rs8003559 | 72808083 | T | C | 1.96E-06 |
| EIA | 14 | rs8022956 | 72808138 | G | A | 1.96E-06 |
| EIA | 14 | rs8003591 | 72808157 | T | C | 1.96E-06 |
| EIA | 14 | rs8022859 | 72808321 | A | G | 2.83E-06 |
| EIA | 14 | chr14:72808625:I | 72808625 | C | CT | 3.22E-06 |
| EIA | 14 | rs2239239 | 72808692 | A | G | 2.85E-06 |
| EIA | 14 | rs2239238 | 72808848 | G | A | 3.98E-06 |
| EIA | 14 | rs2283388 | 72809040 | A | G | 3.95E-06 |
| EIA | 14 | rs929413 | 72810140 | T | C | 4.09E-06 |
| EIA | 14 | rs1812041 | 72810256 | G | A | 2.74E-06 |
| EIA | 14 | chr14:72810344:I | 72810344 | T | TC | 2.75E-06 |
| EIA | 14 | rs2877805 | 72810345 | T | C | 1.89E-06 |
| EIA | 14 | rs2247090 | 72810384 | C | G | 2.74E-06 |
| EIA | 14 | rs886826 | 72810663 | C | T | 2.74E-06 |
| EIA | 14 | rs2239235 | 72811156 | T | C | 2.61E-06 |
| EIA | 14 | rs2681727 | 72811484 | A | C | 1.73E-06 |
| EIA | 14 | rs2681728 | 72811529 | A | G | 6.27E-06 |
| EIA | 14 | rs2681730 | 72811992 | A | G | 2.19E-06 |
| EIA | 14 | rs2681731 | 72812162 | G | A | 2.53E-06 |
| EIA | 14 | rs2681733 | 72812219 | A | G | 2.19E-06 |
| EIA | 14 | rs8016719 | 72812230 | T | A | 3.89E-06 |
| EIA | 14 | rs8016720 | 72812231 | T | A | 3.79E-06 |
| EIA | 14 | rs8015917 | 72812274 | G | A | 2.32E-06 |
| EIA | 14 | rs2681734 | 72812326 | T | G | 1.74E-06 |
| EIA | 14 | rs2681735 | 72812525 | A | G | 1.13E-06 |
| EIA | 14 | rs2681736 | 72812684 | G | T | 2.18E-06 |
| EIA | 14 | rs2529472 | 72812723 | T | C | 3.29E-06 |
| EIA | 14 | rs2681737 | 72812724 | G | A | 3.76E-06 |
| EIA | 14 | rs2529471 | 72813269 | A | C | 1.71E-06 |
| EIA | 14 | rs2529470 | 72813592 | T | C | 1.70E-06 |
| EIA | 14 | rs2529469 | 72813721 | T | C | 1.70E-06 |
| EIA | 14 | rs2239234 | 72813956 | G | A | 1.30E-06 |
| EIA | 14 | rs2239233 | 72814124 | C | A | 1.92E-06 |
| EIA | 14 | rs2239232 | 72814130 | G | A | 1.67E-06 |
| EIA | 14 | rs1356841 | 72814415 | T | C | 2.43E-06 |
| EIA | 14 | rs1356843 | 72814653 | G | T | 1.55E-06 |
| EIA | 14 | rs2681741 | 72814857 | A | G | 9.52E-06 |
| EIA | 14 | chr14:72814877:I | 72814877 | C | CAG | 9.56E-06 |
| EIA | 14 | rs2681743 | 72815145 | T | C | 8.75E-06 |
| EIA | 14 | rs2681744 | 72815284 | C | A | 8.59E-06 |
| EIA | 14 | rs2681745 | 72815405 | T | G | 9.35E-06 |
| EIA | 14 | rs10129799 | 72826071 | A | G | 8.85E-06 |
| EIA | 15 | rs17786786 | 71608619 | A | C | 7.25E-06 |
| EIA | 15 | rs75393192 | 75870551 | C | T | 2.30E-06 |
| EIA | 17 | rs7221929 | 3376188 | A | G | 6.10E-06 |
| EIA | 17 | rs12940665 | 3376519 | T | G | 4.96E-06 |
| EIA | 17 | rs7220982 | 3376682 | G | A | 5.13E-06 |
| EIA | 17 | rs7207788 | 3376885 | T | C | 6.76E-06 |
| EIA | 17 | chr17:3377160:I | 3377160 | C | CTGGAAG | 6.83E-06 |
| EIA | 17 | rs11078450 | 3377396 | C | G | 4.92E-06 |
| EIA | 17 | rs11078451 | 3377743 | G | A | 4.64E-06 |
| EIA | 17 | chr17:3383197:D | 3383197 | GA | G | 2.78E-06 |
| EIA | 17 | rs11651636 | 3383527 | A | G | 2.04E-06 |
| EIA | 17 | rs11656291 | 3383573 | C | T | 2.04E-06 |
| EIA | 17 | chr17:3386126:D | 3386126 | CA | C | 1.93E-06 |
| EIA | 17 | rs62071297 | 3389006 | T | A | 3.20E-06 |
| EIA | 18 | rs55916387 | 31246244 | A | G | 8.30E-06 |
| EIA | 19 | rs62109865 | 13037734 | G | A | 8.25E-06 |
| EIA | 19 | rs6509942 | 30554951 | A | T | 2.19E-06 |
| EIA | 20 | rs75727053 | 36824720 | T | G | 1.37E-08 |
| EIA | 22 | rs422942 | 21423993 | C | G | 8.25E-06 |
| EIA | 22 | rs392358 | 21426113 | A | G | 8.00E-06 |
| EIA | 22 | rs364968 | 21426740 | T | C | 8.38E-06 |
| EIA | 22 | rs403653 | 21427577 | A | G | 5.60E-06 |
| EIA | 22 | rs421166 | 21428523 | G | A | 6.59E-06 |
| EIA | 22 | rs396705 | 21429385 | C | A | 3.99E-06 |
| EIA | 22 | rs400946 | 21431054 | T | C | 2.53E-06 |
| EIA | 22 | rs439792 | 21431181 | C | A | 2.60E-06 |
| Phenotype | Chromosome | rsid | pos | allele_A | allele_B | PCR_frequentist_add_expected_pvalue |
| PCR | 1 | rs34599045 | 1.53E+08 | G | C | 4.46E-06 |
| PCR | 1 | rs12039519 | 1.62E+08 | G | A | 6.53E-06 |
| PCR | 2 | rs72806423 | 47479014 | A | T | 9.74E-06 |
| PCR | 2 | rs10190105 | 47481641 | A | G | 8.52E-06 |
| PCR | 2 | rs12612772 | 47482167 | G | A | 9.30E-06 |
| PCR | 3 | rs6800768 | 24113252 | T | C | 6.40E-06 |
| PCR | 3 | rs4855941 | 1.17E+08 | A | G | 8.55E-06 |
| PCR | 3 | rs7632070 | 1.17E+08 | A | G | 7.51E-06 |
| PCR | 4 | rs12510524 | 8643085 | C | A | 6.51E-06 |
| PCR | 4 | rs1008375 | 17682834 | T | C | 9.43E-06 |
| PCR | 4 | rs56279505 | 1E+08 | T | A | 7.52E-07 |
| PCR | 4 | rs73832804 | 1E+08 | A | G | 3.78E-06 |
| PCR | 4 | rs73832805 | 1E+08 | G | A | 3.82E-06 |
| PCR | 4 | chr4:100312961:D | 1E+08 | AGT | A | 5.04E-06 |
| PCR | 6 | rs11962257 | 8168952 | T | C | 8.55E-06 |
| PCR | 6 | rs11964561 | 8169800 | G | A | 8.25E-06 |
| PCR | 6 | rs6930720 | 8171106 | C | T | 9.75E-06 |
| PCR | 6 | rs9443406 | 78092636 | C | A | 2.95E-06 |
| PCR | 6 | rs7769433 | 78094062 | G | A | 2.95E-06 |
| PCR | 6 | rs6920118 | 78095157 | T | C | 2.99E-06 |
| PCR | 6 | rs1936166 | 78098653 | G | A | 8.70E-07 |
| PCR | 6 | rs1419085 | 78100404 | T | C | 9.34E-07 |
| PCR | 6 | rs1419084 | 78100688 | G | A | 3.07E-06 |
| PCR | 6 | rs1342631 | 78101576 | T | C | 3.07E-06 |
| PCR | 6 | chr6:78102344:D | 78102344 | TC | T | 4.95E-06 |
| PCR | 6 | rs1777822 | 78102380 | G | T | 3.03E-06 |
| PCR | 6 | rs1738535 | 78102499 | C | T | 3.06E-06 |
| PCR | 6 | rs1738534 | 78102549 | T | A | 3.06E-06 |
| PCR | 6 | rs1615713 | 78102849 | T | G | 3.93E-06 |
| PCR | 6 | rs1592315 | 78102854 | A | G | 4.12E-06 |
| PCR | 6 | rs1614775 | 78102964 | C | T | 3.06E-06 |
| PCR | 6 | rs1419083 | 78102988 | C | A | 3.06E-06 |
| PCR | 6 | rs1613126 | 78103116 | G | C | 3.06E-06 |
| PCR | 6 | rs1626310 | 78103283 | G | T | 3.66E-06 |
| PCR | 6 | rs1738532 | 78103562 | C | T | 3.06E-06 |
| PCR | 6 | rs1738531 | 78103588 | T | C | 2.60E-06 |
| PCR | 6 | rs1738530 | 78104417 | T | C | 3.07E-06 |
| PCR | 6 | rs1738529 | 78104655 | T | G | 3.07E-06 |
| PCR | 6 | rs1738528 | 78104711 | A | T | 5.55E-06 |
| PCR | 6 | rs2746595 | 78105177 | A | G | 3.07E-06 |
| PCR | 6 | rs1738527 | 78105318 | T | C | 3.07E-06 |
| PCR | 6 | rs1738526 | 78105486 | C | A | 3.41E-06 |
| PCR | 6 | rs1777820 | 78105741 | G | A | 3.06E-06 |
| PCR | 6 | chr6:78105745:D | 78105745 | AATATTT | A | 2.54E-06 |
| PCR | 6 | rs1777819 | 78105797 | A | G | 3.07E-06 |
| PCR | 6 | rs1738525 | 78105825 | C | T | 3.61E-06 |
| PCR | 6 | chr6:78106368:D | 78106368 | CTGCATGGAT | C | 2.39E-06 |
| PCR | 6 | rs1738524 | 78106449 | T | C | 2.97E-06 |
| PCR | 6 | rs1777818 | 78106537 | C | T | 2.99E-06 |
| PCR | 6 | rs1777817 | 78106600 | G | A | 3.84E-06 |
| PCR | 6 | rs1777815 | 78107052 | A | G | 3.02E-06 |
| PCR | 6 | rs1777814 | 78107195 | C | A | 3.02E-06 |
| PCR | 6 | rs1777813 | 78107322 | G | A | 3.03E-06 |
| PCR | 6 | rs1777812 | 78107344 | T | A | 2.83E-06 |
| PCR | 6 | rs1738523 | 78107599 | C | T | 3.03E-06 |
| PCR | 6 | rs1777811 | 78107827 | T | G | 3.03E-06 |
| PCR | 6 | rs1738522 | 78107854 | T | C | 3.03E-06 |
| PCR | 6 | rs1777810 | 78107896 | C | G | 3.00E-06 |
| PCR | 6 | rs1738521 | 78107959 | G | A | 3.03E-06 |
| PCR | 6 | rs1777809 | 78108355 | G | A | 3.03E-06 |
| PCR | 6 | rs1777808 | 78108624 | A | G | 3.03E-06 |
| PCR | 6 | rs1777807 | 78108747 | A | G | 2.88E-06 |
| PCR | 6 | rs2746594 | 78108825 | G | A | 3.60E-06 |
| PCR | 6 | rs2746593 | 78108829 | G | A | 3.59E-06 |
| PCR | 6 | rs1777806 | 78108843 | A | G | 3.55E-06 |
| PCR | 6 | rs1777805 | 78108859 | A | C | 3.61E-06 |
| PCR | 6 | rs74566120 | 78108958 | C | T | 3.62E-06 |
| PCR | 6 | rs1777803 | 78109222 | A | G | 3.65E-06 |
| PCR | 6 | chr6:78109635:I | 78109635 | T | TA | 3.13E-06 |
| PCR | 6 | rs113832082 | 78109886 | A | G | 3.65E-06 |
| PCR | 6 | rs115040360 | 78109888 | T | C | 8.12E-06 |
| PCR | 6 | rs114186984 | 78109889 | G | A | 8.08E-06 |
| PCR | 6 | rs2746592 | 78110081 | C | T | 3.10E-06 |
| PCR | 6 | chr6:78110141:I | 78110141 | A | AGG | 3.10E-06 |
| PCR | 6 | rs2798523 | 78110498 | A | C | 3.10E-06 |
| PCR | 6 | rs2746591 | 78110556 | C | T | 3.10E-06 |
| PCR | 6 | rs2746590 | 78110692 | G | C | 1.06E-06 |
| PCR | 6 | rs59227895 | 78110943 | T | C | 3.56E-06 |
| PCR | 6 | rs1738519 | 78111780 | C | A | 3.65E-06 |
| PCR | 6 | chr6:78111899:D | 78111899 | GCA | G | 3.65E-06 |
| PCR | 6 | rs1738518 | 78111923 | A | G | 3.70E-06 |
| PCR | 6 | rs1777800 | 78112291 | G | A | 3.65E-06 |
| PCR | 6 | rs2798525 | 78112717 | A | G | 3.65E-06 |
| PCR | 6 | rs2798526 | 78112763 | A | G | 3.65E-06 |
| PCR | 6 | rs1738516 | 78113822 | G | C | 2.96E-06 |
| PCR | 6 | rs196670 | 80113595 | G | A | 3.32E-06 |
| PCR | 6 | rs9448722 | 80168035 | C | G | 3.68E-06 |
| PCR | 7 | rs77689838 | 67591039 | C | T | 5.94E-06 |
| PCR | 7 | chr7:98492246:I | 98492246 | G | GT | 5.61E-06 |
| PCR | 7 | chr7:98495304:I | 98495304 | G | GTT | 7.58E-06 |
| PCR | 7 | rs2237598 | 98540123 | T | C | 7.02E-06 |
| PCR | 7 | rs6967970 | 98544342 | T | G | 9.69E-06 |
| PCR | 7 | rs4727424 | 98545103 | G | A | 9.62E-06 |
| PCR | 7 | rs7779292 | 98548842 | C | G | 6.29E-06 |
| PCR | 7 | rs3815232 | 98551152 | C | T | 8.75E-06 |
| PCR | 7 | rs41646 | 98571011 | G | C | 8.45E-06 |
| PCR | 8 | rs2930345 | 3734727 | G | C | 3.90E-06 |
| PCR | 8 | rs17067840 | 3736282 | G | C | 3.25E-06 |
| PCR | 8 | rs10090300 | 3739619 | T | C | 4.57E-06 |
| PCR | 8 | rs10113221 | 3743704 | T | G | 2.33E-06 |
| PCR | 8 | rs2688402 | 3763463 | C | T | 8.39E-06 |
| PCR | 8 | rs143889815 | 22928311 | C | T | 1.63E-06 |
| PCR | 8 | rs57302454 | 22938661 | G | C | 5.74E-07 |
| PCR | 8 | rs3892512 | 53010163 | G | C | 9.86E-06 |
| PCR | 8 | rs13261812 | 1.21E+08 | T | A | 3.40E-06 |
| PCR | 8 | rs2305598 | 1.21E+08 | T | C | 1.80E-06 |
| PCR | 8 | rs7013781 | 1.21E+08 | G | A | 9.04E-07 |
| PCR | 8 | rs7013555 | 1.21E+08 | A | G | 1.01E-06 |
| PCR | 8 | rs7018328 | 1.21E+08 | C | G | 1.18E-06 |
| PCR | 8 | rs6980799 | 1.21E+08 | G | A | 1.29E-06 |
| PCR | 8 | rs2305600 | 1.21E+08 | T | C | 1.05E-06 |
| PCR | 8 | rs2054148 | 1.21E+08 | C | T | 8.61E-07 |
| PCR | 8 | rs10955961 | 1.21E+08 | T | C | 2.76E-07 |
| PCR | 8 | rs10955962 | 1.21E+08 | A | T | 4.98E-07 |
| PCR | 8 | rs957694 | 1.21E+08 | T | C | 1.44E-06 |
| PCR | 8 | rs957695 | 1.21E+08 | A | T | 1.61E-06 |
| PCR | 8 | rs957696 | 1.21E+08 | A | G | 1.60E-06 |
| PCR | 8 | rs3765062 | 1.21E+08 | G | A | 1.13E-06 |
| PCR | 8 | rs2875940 | 1.21E+08 | C | T | 4.67E-06 |
| PCR | 8 | rs2034841 | 1.21E+08 | T | C | 1.12E-06 |
| PCR | 8 | rs2034842 | 1.21E+08 | T | C | 6.36E-07 |
| PCR | 8 | rs2219246 | 1.21E+08 | A | C | 6.18E-06 |
| PCR | 8 | rs12547117 | 1.21E+08 | C | T | 6.45E-06 |
| PCR | 8 | rs12542144 | 1.21E+08 | A | G | 6.23E-06 |
| PCR | 8 | rs10808508 | 1.21E+08 | C | T | 4.18E-07 |
| PCR | 8 | rs4870723 | 1.21E+08 | A | C | 7.51E-06 |
| PCR | 8 | rs4870724 | 1.21E+08 | T | G | 1.36E-06 |
| PCR | 8 | rs7387094 | 1.21E+08 | A | G | 4.89E-07 |
| PCR | 8 | rs7387373 | 1.21E+08 | C | T | 8.38E-07 |
| PCR | 8 | rs56159702 | 1.21E+08 | T | C | 3.79E-07 |
| PCR | 8 | rs58829748 | 1.21E+08 | C | T | 7.19E-07 |
| PCR | 8 | rs12543412 | 1.21E+08 | T | C | 9.08E-06 |
| PCR | 8 | rs11773991 | 1.21E+08 | T | C | 1.83E-06 |
| PCR | 8 | chr8:121234238:I | 1.21E+08 | G | GT | 4.50E-07 |
| PCR | 8 | rs6989074 | 1.21E+08 | G | A | 5.03E-07 |
| PCR | 10 | rs12255911 | 13685247 | T | C | 7.37E-06 |
| PCR | 10 | rs56161216 | 13688798 | A | G | 6.39E-06 |
| PCR | 10 | rs74123095 | 13698078 | T | A | 7.35E-06 |
| PCR | 10 | rs11818229 | 13700805 | A | C | 8.39E-06 |
| PCR | 10 | rs58180147 | 69943634 | C | T | 6.70E-06 |
| PCR | 11 | rs4408325 | 1.25E+08 | T | A | 1.45E-07 |
| PCR | 11 | rs4936963 | 1.25E+08 | A | C | 1.57E-06 |
| PCR | 14 | rs34310550 | 38912395 | C | T | 8.57E-06 |
| PCR | 16 | rs117559033 | 25944544 | C | T | 2.08E-06 |
| PCR | 18 | rs116303449 | 32956931 | A | G | 5.93E-07 |
| PCR | 21 | rs12483240 | 26829777 | C | T | 2.95E-06 |
| PCR | 21 | rs2829751 | 26829787 | G | C | 3.04E-06 |
| PCR | 21 | rs79728973 | 33209503 | G | A | 9.90E-06 |
| PCR | 21 | rs77292666 | 33214050 | G | A | 5.87E-06 |
| PCR | 22 | rs16997854 | 37562146 | C | T | 6.58E-06 |
| PCR | 22 | rs12171227 | 37562467 | G | A | 6.79E-06 |
| PCR | 22 | rs12167757 | 37567490 | G | A | 5.76E-06 |
| PCR | 22 | rs2160908 | 37586792 | C | T | 4.74E-06 |
| Phenotype | Chromsome | rsid | pos | allele_A | allele_B | PR_frequentist_add_expected_pvalue |
| PR | 1 | rs3011925 | 7721360 | G | A | 3.44E-06 |
| PR | 1 | rs111724808 | 22737946 | T | G | 3.96E-07 |
| PR | 1 | rs113487266 | 22737951 | T | C | 4.03E-07 |
| PR | 1 | rs111245681 | 22737964 | T | C | 8.71E-07 |
| PR | 1 | rs76652604 | 22738033 | G | T | 4.33E-07 |
| PR | 1 | rs1776421 | 53884457 | C | T | 6.24E-06 |
| PR | 1 | rs114147144 | 54443865 | T | C | 2.81E-06 |
| PR | 1 | rs11810300 | 57654660 | C | T | 9.01E-06 |
| PR | 1 | rs1394397 | 57656611 | A | G | 2.92E-06 |
| PR | 1 | rs77177281 | 98471702 | A | G | 2.44E-06 |
| PR | 1 | rs631288 | 1.47E+08 | C | T | 2.63E-06 |
| PR | 2 | rs55678912 | 16191545 | C | T | 6.50E-09 |
| PR | 2 | rs7569161 | 16193273 | A | G | 3.93E-08 |
| PR | 2 | rs72770595 | 16196085 | A | G | 6.04E-08 |
| PR | 2 | rs72770598 | 16197939 | T | A | 4.58E-08 |
| PR | 2 | rs72770600 | 16198363 | T | C | 4.58E-08 |
| PR | 2 | rs72770601 | 16198672 | C | T | 5.94E-08 |
| PR | 2 | rs7608858 | 16201566 | C | T | 1.51E-07 |
| PR | 2 | rs78556816 | 68386026 | C | T | 1.23E-06 |
| PR | 2 | rs72909550 | 71526524 | G | T | 9.86E-06 |
| PR | 2 | rs7609266 | 71530558 | G | C | 6.35E-06 |
| PR | 2 | rs17692517 | 71563549 | T | G | 8.67E-06 |
| PR | 2 | rs72909586 | 71567349 | T | C | 5.70E-06 |
| PR | 2 | rs7557781 | 71613914 | A | G | 9.72E-06 |
| PR | 2 | chr2:71687004:D | 71687004 | TA | T | 9.55E-06 |
| PR | 2 | rs56988800 | 71688118 | C | A | 1.33E-07 |
| PR | 2 | rs80255579 | 71690472 | C | A | 5.41E-06 |
| PR | 2 | rs74836064 | 71690528 | G | A | 1.64E-06 |
| PR | 2 | rs4362586 | 71690647 | T | C | 1.69E-06 |
| PR | 2 | rs4542877 | 71690715 | G | A | 1.71E-06 |
| PR | 2 | rs4629186 | 71690800 | C | T | 1.75E-06 |
| PR | 2 | rs74776753 | 71690944 | G | A | 1.78E-06 |
| PR | 2 | rs75252201 | 71691063 | G | A | 1.80E-06 |
| PR | 2 | rs111519406 | 71691099 | G | A | 1.80E-06 |
| PR | 2 | rs78624083 | 71691457 | G | C | 1.88E-06 |
| PR | 2 | rs78130680 | 71691604 | C | T | 9.31E-06 |
| PR | 2 | rs4538230 | 71691896 | T | A | 1.90E-06 |
| PR | 2 | rs4322872 | 71691899 | A | G | 1.90E-06 |
| PR | 2 | rs4547553 | 71691940 | G | A | 1.91E-06 |
| PR | 2 | rs4353676 | 71691942 | A | G | 1.89E-06 |
| PR | 2 | rs72896814 | 71692152 | A | G | 1.11E-06 |
| PR | 2 | rs80137915 | 71692195 | G | A | 1.89E-06 |
| PR | 2 | rs79523511 | 71692883 | C | G | 1.82E-07 |
| PR | 2 | rs35349447 | 1.57E+08 | T | C | 3.93E-06 |
| PR | 2 | chr2:156576593:D | 1.57E+08 | GT | G | 9.14E-06 |
| PR | 2 | rs12373770 | 1.57E+08 | T | A | 7.51E-06 |
| PR | 2 | rs2350817 | 1.57E+08 | T | A | 4.29E-06 |
| PR | 2 | rs4664774 | 1.57E+08 | T | G | 2.86E-06 |
| PR | 2 | chr2:156582187:I | 1.57E+08 | A | AAATTT | 5.87E-06 |
| PR | 2 | rs13385378 | 2.16E+08 | A | G | 9.15E-06 |
| PR | 2 | rs10210200 | 2.16E+08 | G | A | 1.61E-06 |
| PR | 3 | rs76090503 | 1129819 | G | A | 4.04E-07 |
| PR | 3 | rs3902530 | 1299185 | A | G | 4.31E-06 |
| PR | 3 | rs9815195 | 1301130 | C | A | 7.41E-07 |
| PR | 3 | rs9834544 | 1301197 | A | G | 7.03E-06 |
| PR | 3 | rs3872629 | 1304868 | A | C | 5.29E-06 |
| PR | 3 | rs9840225 | 25181030 | G | A | 5.78E-06 |
| PR | 3 | chr3:75669349:D | 75669349 | AGTCAG | A | 9.77E-06 |
| PR | 3 | rs188655146 | 86544386 | T | G | 8.82E-06 |
| PR | 3 | rs7640139 | 1.24E+08 | C | T | 8.40E-06 |
| PR | 3 | rs2332737 | 1.24E+08 | G | A | 8.76E-06 |
| PR | 3 | rs6438827 | 1.24E+08 | G | A | 9.58E-06 |
| PR | 3 | rs4678082 | 1.24E+08 | A | G | 3.04E-06 |
| PR | 3 | rs1920616 | 1.24E+08 | G | A | 2.58E-06 |
| PR | 3 | rs6794106 | 1.24E+08 | G | C | 2.85E-06 |
| PR | 3 | rs13087591 | 1.24E+08 | G | A | 3.54E-06 |
| PR | 3 | rs13067260 | 1.24E+08 | T | G | 1.87E-06 |
| PR | 3 | rs1920625 | 1.24E+08 | C | T | 2.48E-06 |
| PR | 3 | rs114542799 | 1.26E+08 | G | A | 4.76E-06 |
| PR | 3 | rs1199338 | 1.38E+08 | A | C | 9.06E-06 |
| PR | 3 | rs1199337 | 1.38E+08 | G | C | 9.16E-06 |
| PR | 3 | rs185244 | 1.38E+08 | C | T | 8.94E-06 |
| PR | 3 | rs181877 | 1.38E+08 | A | G | 8.06E-06 |
| PR | 3 | rs1199340 | 1.38E+08 | G | A | 1.51E-06 |
| PR | 3 | rs1678443 | 1.38E+08 | C | A | 1.48E-06 |
| PR | 3 | rs774009 | 1.38E+08 | G | A | 4.70E-06 |
| PR | 3 | rs295490 | 1.39E+08 | C | T | 8.08E-06 |
| PR | 3 | rs116465281 | 1.43E+08 | C | T | 3.52E-06 |
| PR | 3 | rs183670238 | 1.44E+08 | T | C | 6.66E-06 |
| PR | 3 | chr3:168234898:D | 1.68E+08 | TTGG | T | 6.37E-06 |
| PR | 3 | rs16852382 | 1.68E+08 | A | G | 2.04E-06 |
| PR | 3 | rs6782264 | 1.68E+08 | C | T | 1.83E-06 |
| PR | 3 | rs73878922 | 1.68E+08 | C | T | 2.08E-06 |
| PR | 3 | rs115965187 | 1.79E+08 | C | T | 6.03E-06 |
| PR | 4 | rs114133078 | 15354385 | T | C | 2.20E-07 |
| PR | 4 | rs75900745 | 1.38E+08 | A | G | 1.67E-06 |
| PR | 5 | rs77202753 | 9619864 | C | T | 7.55E-07 |
| PR | 5 | rs20476 | 11366157 | C | T | 4.23E-06 |
| PR | 5 | chr5:13260590:D | 13260590 | GA | G | 1.21E-06 |
| PR | 5 | rs114846327 | 36436579 | A | G | 2.28E-06 |
| PR | 5 | rs73117804 | 75649280 | C | T | 9.34E-06 |
| PR | 5 | rs55988457 | 96949073 | T | C | 5.62E-08 |
| PR | 5 | rs78979090 | 1.2E+08 | C | G | 3.16E-06 |
| PR | 5 | rs11948803 | 1.2E+08 | A | T | 3.85E-06 |
| PR | 5 | rs77726200 | 1.2E+08 | T | G | 4.85E-06 |
| PR | 5 | rs919708 | 1.2E+08 | T | C | 4.96E-06 |
| PR | 5 | rs115909428 | 1.2E+08 | T | C | 5.34E-06 |
| PR | 5 | rs17146503 | 1.2E+08 | T | C | 3.79E-06 |
| PR | 5 | rs139749488 | 1.2E+08 | T | C | 4.33E-06 |
| PR | 5 | rs114266291 | 1.21E+08 | A | C | 9.56E-06 |
| PR | 5 | rs186263310 | 1.28E+08 | A | G | 5.16E-06 |
| PR | 5 | rs115545556 | 1.31E+08 | C | T | 4.73E-07 |
| PR | 5 | rs152447 | 1.42E+08 | A | T | 9.27E-06 |
| PR | 5 | chr5:141923528:D | 1.42E+08 | TTCC | T | 6.65E-06 |
| PR | 5 | rs152438 | 1.42E+08 | C | T | 8.74E-06 |
| PR | 5 | rs152439 | 1.42E+08 | T | C | 3.57E-06 |
| PR | 5 | chr5:152308277:I | 1.52E+08 | C | CT | 3.34E-07 |
| PR | 5 | rs75380573 | 1.52E+08 | G | A | 1.37E-06 |
| PR | 5 | rs79210417 | 1.52E+08 | T | C | 1.37E-06 |
| PR | 5 | rs75703928 | 1.52E+08 | G | A | 1.39E-06 |
| PR | 5 | rs113013679 | 1.52E+08 | G | A | 1.41E-06 |
| PR | 5 | rs112895793 | 1.52E+08 | T | C | 1.32E-06 |
| PR | 5 | rs78040934 | 1.52E+08 | G | A | 1.44E-06 |
| PR | 5 | rs78988005 | 1.52E+08 | T | C | 1.24E-06 |
| PR | 5 | rs192943752 | 1.52E+08 | T | C | 1.44E-06 |
| PR | 5 | rs79872771 | 1.52E+08 | T | A | 5.76E-06 |
| PR | 5 | chr5:152394641:I | 1.52E+08 | A | AG | 1.44E-06 |
| PR | 5 | rs59900286 | 1.52E+08 | A | G | 1.44E-06 |
| PR | 5 | rs115143232 | 1.52E+08 | C | T | 1.44E-06 |
| PR | 5 | chr5:152396902:D | 1.52E+08 | AAT | A | 1.45E-06 |
| PR | 5 | rs56994551 | 1.52E+08 | T | A | 1.89E-06 |
| PR | 5 | rs76768621 | 1.52E+08 | C | A | 1.45E-06 |
| PR | 5 | rs111911908 | 1.52E+08 | C | T | 2.80E-06 |
| PR | 5 | rs181619037 | 1.52E+08 | G | A | 1.46E-06 |
| PR | 5 | rs143525730 | 1.52E+08 | T | C | 2.81E-06 |
| PR | 5 | rs9716680 | 1.52E+08 | C | T | 1.54E-06 |
| PR | 5 | rs76483517 | 1.52E+08 | C | T | 5.18E-07 |
| PR | 5 | rs79228075 | 1.52E+08 | C | T | 1.47E-06 |
| PR | 5 | rs114440438 | 1.52E+08 | C | T | 1.47E-06 |
| PR | 5 | rs75173757 | 1.52E+08 | T | G | 1.49E-06 |
| PR | 5 | rs113356988 | 1.52E+08 | C | A | 1.51E-06 |
| PR | 5 | rs111685453 | 1.52E+08 | G | A | 1.51E-06 |
| PR | 5 | rs75515828 | 1.52E+08 | A | G | 1.51E-06 |
| PR | 5 | chr5:152425113:D | 1.52E+08 | AAC | A | 2.66E-06 |
| PR | 5 | rs146717151 | 1.52E+08 | A | C | 1.52E-06 |
| PR | 5 | rs56760618 | 1.52E+08 | G | A | 1.52E-06 |
| PR | 5 | rs61419327 | 1.52E+08 | A | G | 1.52E-06 |
| PR | 5 | rs57884840 | 1.52E+08 | C | T | 1.52E-06 |
| PR | 5 | rs78188846 | 1.52E+08 | G | T | 1.53E-06 |
| PR | 5 | rs80314334 | 1.52E+08 | A | G | 1.54E-06 |
| PR | 5 | rs78748990 | 1.52E+08 | T | C | 1.91E-06 |
| PR | 5 | rs73798901 | 1.52E+08 | A | G | 2.60E-06 |
| PR | 5 | rs73798902 | 1.52E+08 | C | T | 2.70E-06 |
| PR | 5 | rs73802005 | 1.52E+08 | T | C | 2.12E-06 |
| PR | 5 | rs73802006 | 1.52E+08 | A | T | 4.78E-06 |
| PR | 5 | rs73802034 | 1.52E+08 | A | G | 6.80E-06 |
| PR | 5 | rs73802035 | 1.52E+08 | A | G | 9.34E-06 |
| PR | 5 | rs61056587 | 1.52E+08 | G | T | 9.52E-06 |
| PR | 5 | rs73798703 | 1.52E+08 | T | C | 9.24E-06 |
| PR | 5 | rs57917647 | 1.53E+08 | C | T | 1.51E-06 |
| PR | 5 | rs74717972 | 1.58E+08 | C | T | 8.49E-06 |
| PR | 5 | rs75248416 | 1.58E+08 | G | C | 8.51E-06 |
| PR | 5 | rs76457712 | 1.58E+08 | C | T | 8.48E-06 |
| PR | 5 | rs74544835 | 1.58E+08 | C | A | 8.48E-06 |
| PR | 5 | rs75897066 | 1.58E+08 | G | A | 8.48E-06 |
| PR | 5 | rs75184948 | 1.58E+08 | G | A | 8.48E-06 |
| PR | 5 | rs75999723 | 1.58E+08 | G | T | 8.48E-06 |
| PR | 5 | rs74918733 | 1.58E+08 | C | T | 8.48E-06 |
| PR | 5 | rs76180878 | 1.58E+08 | T | C | 8.48E-06 |
| PR | 5 | rs74620148 | 1.58E+08 | C | A | 6.21E-06 |
| PR | 5 | rs78409910 | 1.58E+08 | G | A | 8.48E-06 |
| PR | 5 | rs76406105 | 1.58E+08 | T | C | 8.48E-06 |
| PR | 5 | rs77308452 | 1.58E+08 | G | T | 8.48E-06 |
| PR | 5 | rs112125540 | 1.58E+08 | G | A | 8.48E-06 |
| PR | 5 | rs76404868 | 1.58E+08 | C | T | 8.48E-06 |
| PR | 5 | rs111874800 | 1.58E+08 | C | T | 8.80E-06 |
| PR | 5 | rs187361198 | 1.58E+08 | T | A | 6.37E-06 |
| PR | 5 | rs76709770 | 1.58E+08 | G | T | 8.52E-06 |
| PR | 6 | rs76439045 | 27411729 | A | C | 5.06E-08 |
| PR | 6 | rs115842765 | 43193318 | G | T | 3.10E-06 |
| PR | 6 | rs114326925 | 43193320 | A | G | 3.10E-06 |
| PR | 6 | rs61018535 | 43449230 | C | A | 1.53E-07 |
| PR | 6 | rs9462897 | 43454962 | A | G | 3.83E-06 |
| PR | 6 | rs116755262 | 44455133 | A | G | 7.49E-07 |
| PR | 6 | rs114761004 | 44457273 | G | A | 1.00E-06 |
| PR | 6 | rs115317167 | 44457274 | C | T | 8.55E-07 |
| PR | 6 | chr6:87600614:I | 87600614 | T | TGC | 1.57E-07 |
| PR | 6 | rs7341237 | 88032390 | G | C | 2.20E-06 |
| PR | 6 | rs9444586 | 88881642 | G | C | 5.66E-06 |
| PR | 6 | rs9450903 | 88882193 | A | G | 5.78E-06 |
| PR | 6 | rs9450904 | 88882493 | C | T | 5.84E-06 |
| PR | 6 | rs9450906 | 88883324 | C | T | 5.62E-06 |
| PR | 6 | rs9450907 | 88883522 | C | A | 5.67E-06 |
| PR | 6 | rs11966501 | 88883547 | T | A | 5.68E-06 |
| PR | 6 | rs11968764 | 88883569 | A | C | 5.69E-06 |
| PR | 6 | rs11963892 | 88883571 | G | A | 6.14E-06 |
| PR | 6 | rs9444588 | 88884166 | C | A | 5.84E-06 |
| PR | 6 | chr6:102155407:D | 1.02E+08 | GTGTGTATATA | G | 3.81E-07 |
| PR | 7 | chr7:1156559:I | 1156559 | A | AC | 8.10E-06 |
| PR | 7 | rs2713339 | 9548713 | C | A | 8.85E-06 |
| PR | 7 | rs2709057 | 9548858 | A | G | 1.00E-05 |
| PR | 7 | rs12702919 | 9550572 | A | C | 4.91E-06 |
| PR | 7 | rs10237257 | 9553854 | G | A | 9.48E-06 |
| PR | 7 | rs4548084 | 9559594 | G | C | 5.48E-06 |
| PR | 7 | rs10452828 | 9559709 | T | G | 5.47E-06 |
| PR | 7 | rs2068399 | 14228703 | G | A | 8.55E-06 |
| PR | 7 | rs61584435 | 18717509 | T | G | 4.15E-06 |
| PR | 7 | rs73313346 | 18723232 | T | C | 4.00E-06 |
| PR | 7 | rs59623110 | 20678749 | T | C | 2.66E-07 |
| PR | 7 | rs73276602 | 20681850 | A | C | 9.88E-08 |
| PR | 7 | rs12019358 | 41275504 | C | T | 4.69E-06 |
| PR | 7 | rs7806994 | 56139505 | A | T | 6.49E-06 |
| PR | 7 | chr7:75365381:D | 75365381 | CT | C | 2.14E-07 |
| PR | 7 | rs113527903 | 77838510 | C | G | 2.96E-06 |
| PR | 7 | rs114137957 | 87374869 | T | G | 9.63E-07 |
| PR | 7 | rs6966264 | 87543037 | C | G | 4.07E-06 |
| PR | 7 | rs2110483 | 93880093 | T | C | 7.32E-06 |
| PR | 7 | rs115744676 | 94061404 | G | T | 6.91E-09 |
| PR | 7 | rs116187617 | 94061601 | C | A | 1.23E-07 |
| PR | 7 | chr7:101117013:D | 1.01E+08 | AG | A | 7.49E-06 |
| PR | 7 | rs73409616 | 1.01E+08 | C | T | 6.64E-06 |
| PR | 7 | chr7:101118545:D | 1.01E+08 | TCTGTGTC | T | 4.07E-06 |
| PR | 7 | rs6967098 | 1.01E+08 | G | A | 9.11E-06 |
| PR | 7 | rs10241622 | 1.01E+08 | C | T | 8.77E-06 |
| PR | 7 | rs73712171 | 1.01E+08 | G | T | 6.50E-06 |
| PR | 7 | rs113727105 | 1.01E+08 | G | A | 9.27E-06 |
| PR | 7 | rs17157913 | 1.1E+08 | T | C | 2.11E-06 |
| PR | 7 | rs2074114 | 1.11E+08 | T | C | 2.80E-06 |
| PR | 7 | rs79274420 | 1.15E+08 | A | G | 4.79E-06 |
| PR | 7 | rs10274759 | 1.15E+08 | A | G | 3.00E-06 |
| PR | 7 | rs147347909 | 1.16E+08 | T | C | 8.57E-06 |
| PR | 7 | rs6959106 | 1.16E+08 | T | C | 1.94E-06 |
| PR | 7 | rs11974088 | 1.16E+08 | C | T | 6.77E-06 |
| PR | 7 | rs7802124 | 1.16E+08 | T | C | 1.69E-06 |
| PR | 7 | rs2052106 | 1.16E+08 | A | G | 4.23E-06 |
| PR | 7 | rs11979486 | 1.16E+08 | T | G | 3.02E-06 |
| PR | 7 | rs6976011 | 1.16E+08 | C | T | 4.22E-06 |
| PR | 7 | rs17869724 | 1.27E+08 | C | T | 1.96E-06 |
| PR | 7 | rs17866046 | 1.27E+08 | A | G | 7.00E-06 |
| PR | 7 | rs17866467 | 1.27E+08 | G | A | 4.48E-06 |
| PR | 7 | rs28947798 | 1.27E+08 | G | A | 4.34E-06 |
| PR | 7 | rs28947799 | 1.27E+08 | G | T | 4.33E-06 |
| PR | 7 | rs28947801 | 1.27E+08 | G | A | 4.30E-06 |
| PR | 7 | rs74789234 | 1.27E+08 | A | C | 3.89E-06 |
| PR | 8 | rs75609241 | 29082285 | G | A | 4.53E-07 |
| PR | 8 | rs79076257 | 1.1E+08 | T | C | 1.50E-07 |
| PR | 8 | rs78908257 | 1.1E+08 | T | C | 3.79E-06 |
| PR | 9 | rs117347249 | 81368654 | A | G | 2.98E-06 |
| PR | 9 | rs140141171 | 82160138 | G | C | 9.70E-06 |
| PR | 9 | rs11138290 | 82163313 | C | T | 2.43E-06 |
| PR | 9 | rs35775808 | 1.02E+08 | A | G | 7.18E-06 |
| PR | 10 | rs79619559 | 2776698 | A | T | 3.48E-06 |
| PR | 10 | rs114088342 | 35918232 | A | G | 1.75E-06 |
| PR | 10 | rs113808744 | 71749675 | T | C | 4.24E-06 |
| PR | 10 | rs28663309 | 1.03E+08 | G | A | 9.92E-06 |
| PR | 11 | rs73469144 | 21504900 | A | C | 6.29E-06 |
| PR | 11 | rs78015633 | 40876586 | T | C | 9.27E-06 |
| PR | 11 | rs78972067 | 70058419 | C | T | 7.87E-06 |
| PR | 12 | rs9332958 | 16519263 | A | G | 4.53E-06 |
| PR | 12 | rs142897702 | 27739570 | C | G | 9.57E-06 |
| PR | 12 | rs116975820 | 42722908 | T | G | 5.05E-06 |
| PR | 12 | rs76573623 | 1.14E+08 | A | G | 5.69E-06 |
| PR | 12 | rs76270203 | 1.14E+08 | A | C | 8.68E-07 |
| PR | 12 | rs75753760 | 1.14E+08 | C | T | 3.28E-06 |
| PR | 12 | rs78852656 | 1.2E+08 | G | A | 3.90E-08 |
| PR | 13 | rs149773673 | 1.05E+08 | G | A | 4.18E-06 |
| PR | 15 | rs2654155 | 31941218 | C | T | 7.57E-06 |
| PR | 15 | rs192142097 | 31986243 | A | G | 4.02E-06 |
| PR | 15 | chr15:32006725:I | 32006725 | A | AT | 8.64E-06 |
| PR | 15 | chr15:76777214:I | 76777214 | C | CT | 1.71E-06 |
| PR | 16 | rs184822539 | 10593203 | T | G | 6.55E-06 |
| PR | 17 | rs59403466 | 2785895 | T | G | 6.38E-07 |
| PR | 17 | rs117364231 | 32306423 | C | T | 9.68E-08 |
| PR | 17 | rs75041531 | 38393151 | A | C | 9.42E-07 |
| PR | 17 | rs191392302 | 39953961 | T | G | 3.18E-08 |
| PR | 17 | chr17:41073503:I | 41073503 | G | GA | 6.04E-06 |
| PR | 17 | rs188757965 | 41119569 | T | G | 8.93E-06 |
| PR | 17 | rs57166100 | 62506280 | T | C | 3.22E-06 |
| PR | 17 | rs77325336 | 66625794 | C | T | 3.52E-06 |
| PR | 18 | chr18:797463:I | 797463 | C | CA | 3.92E-06 |
| PR | 18 | rs11664027 | 807368 | T | G | 2.46E-07 |
| PR | 18 | rs1146075 | 1366301 | C | T | 5.21E-07 |
| PR | 18 | rs2345595 | 1818385 | A | C | 3.95E-06 |
| PR | 18 | rs6505987 | 1977713 | G | T | 3.08E-08 |
| PR | 18 | chr18:2227374:I | 2227374 | G | GA | 3.64E-06 |
| PR | 18 | rs8084727 | 2452129 | T | A | 5.79E-06 |
| PR | 18 | rs3914785 | 2452177 | A | G | 2.28E-06 |
| PR | 18 | chr18:2510020:I | 2510020 | T | TA | 7.30E-06 |
| PR | 18 | rs182046301 | 2614475 | A | G | 2.41E-06 |
| PR | 18 | rs4533365 | 2645746 | A | G | 3.18E-06 |
| PR | 18 | rs28877609 | 2865505 | G | A | 2.90E-06 |
| PR | 18 | rs8087073 | 2967845 | A | C | 1.08E-09 |
| PR | 18 | rs115445970 | 3056424 | T | A | 9.96E-06 |
| PR | 18 | rs58882377 | 3465132 | A | G | 8.08E-06 |
| PR | 18 | rs12185468 | 8479685 | C | T | 3.01E-06 |
| PR | 18 | rs79285331 | 61145707 | C | T | 2.64E-06 |
| PR | 18 | rs73963337 | 64697353 | C | G | 2.13E-07 |
| PR | 18 | rs28708459 | 64698046 | T | A | 7.86E-07 |
| PR | 18 | rs28562244 | 64698137 | C | T | 7.79E-07 |
| PR | 18 | chr18:64699809:D | 64699809 | AATTT | A | 1.07E-07 |
| PR | 18 | rs73963343 | 64699974 | C | T | 8.86E-08 |
| PR | 19 | rs147615524 | 1205889 | C | T | 1.51E-06 |
| PR | 19 | rs35967093 | 4883741 | A | T | 6.15E-06 |
| PR | 20 | rs3176130 | 23031656 | C | T | 1.54E-07 |
| PR | 21 | rs11909679 | 32304057 | G | A | 9.40E-06 |
| PR | 21 | rs11909638 | 32304174 | A | T | 8.00E-06 |
| PR | 21 | rs8134605 | 32304266 | G | C | 7.85E-07 |
| PR | 22 | rs79384503 | 22047969 | C | T | 2.05E-06 |
| Phenotype | Chromosome | rsid | pos | allele_A | allele_B | QRS_frequentist_add_expected_pvalue |
| QRS | 1 | rs28829049 | 19349281 | C | T | 6.46E-06 |
| QRS | 1 | rs1146509 | 95029581 | G | T | 6.05E-06 |
| QRS | 1 | rs1376359 | 1.03E+08 | T | C | 9.36E-06 |
| QRS | 1 | rs73028893 | 1.67E+08 | A | G | 5.59E-06 |
| QRS | 2 | rs3749056 | 40484945 | T | C | 3.64E-06 |
| QRS | 3 | rs155206 | 6562331 | A | G | 2.32E-06 |
| QRS | 3 | rs409974 | 21447336 | G | A | 8.95E-06 |
| QRS | 3 | rs7647212 | 1.42E+08 | G | A | 6.73E-06 |
| QRS | 3 | rs7647497 | 1.42E+08 | G | A | 7.07E-06 |
| QRS | 3 | rs9844137 | 1.42E+08 | T | A | 4.53E-06 |
| QRS | 3 | rs9816128 | 1.42E+08 | C | T | 4.53E-06 |
| QRS | 3 | rs9816275 | 1.42E+08 | C | G | 4.53E-06 |
| QRS | 3 | rs7633021 | 1.42E+08 | T | C | 4.38E-06 |
| QRS | 3 | rs9826463 | 1.42E+08 | C | G | 4.16E-06 |
| QRS | 3 | rs56383228 | 1.42E+08 | T | C | 4.60E-06 |
| QRS | 3 | rs6440097 | 1.42E+08 | G | C | 4.42E-06 |
| QRS | 3 | rs28709617 | 1.42E+08 | C | G | 4.41E-06 |
| QRS | 3 | rs28378300 | 1.42E+08 | T | C | 4.40E-06 |
| QRS | 3 | rs28647471 | 1.42E+08 | A | C | 4.40E-06 |
| QRS | 3 | rs4398471 | 1.42E+08 | A | G | 4.39E-06 |
| QRS | 3 | rs9868309 | 1.42E+08 | A | G | 4.37E-06 |
| QRS | 3 | rs2140433 | 1.42E+08 | C | G | 4.85E-06 |
| QRS | 3 | chr3:142343788:D | 1.42E+08 | GGAT | G | 6.87E-06 |
| QRS | 4 | rs78792420 | 24778280 | T | G | 1.85E-06 |
| QRS | 5 | rs12109307 | 1.21E+08 | T | C | 1.81E-06 |
| QRS | 5 | rs76616598 | 1.21E+08 | T | C | 2.37E-06 |
| QRS | 5 | chr5:163228838:I | 1.63E+08 | T | TA | 1.36E-06 |
| QRS | 6 | rs182503338 | 34582274 | T | G | 7.95E-07 |
| QRS | 7 | rs1919796 | 9323220 | G | A | 5.97E-06 |
| QRS | 7 | rs1852612 | 45400895 | C | T | 8.28E-06 |
| QRS | 7 | rs77909595 | 95700008 | C | T | 2.44E-06 |
| QRS | 8 | rs4242337 | 1.24E+08 | G | A | 9.70E-06 |
| QRS | 9 | rs378628 | 10054257 | T | C | 7.01E-06 |
| QRS | 9 | rs447578 | 10054522 | T | C | 6.46E-06 |
| QRS | 10 | chr10:14886486:D | 14886486 | CG | C | 1.35E-06 |
| QRS | 10 | rs7894284 | 14894901 | T | C | 6.09E-06 |
| QRS | 10 | rs11012167 | 20862401 | C | T | 5.60E-06 |
| QRS | 10 | rs16920681 | 20864666 | A | G | 5.78E-06 |
| QRS | 10 | rs72786268 | 25770025 | G | C | 3.81E-07 |
| QRS | 10 | rs12251514 | 52259794 | A | C | 5.27E-06 |
| QRS | 11 | rs79243044 | 5556055 | T | C | 8.62E-06 |
| QRS | 11 | rs10769782 | 7390571 | G | C | 6.28E-06 |
| QRS | 11 | rs10769783 | 7396191 | A | G | 2.07E-07 |
| QRS | 11 | chr11:7401829:I | 7401829 | T | TA | 4.59E-06 |
| QRS | 11 | rs72846013 | 7408743 | G | A | 5.64E-06 |
| QRS | 11 | rs145725684 | 25214237 | A | G | 6.56E-06 |
| QRS | 11 | rs112328818 | 25215421 | A | G | 7.56E-06 |
| QRS | 11 | rs79996542 | 25215979 | T | A | 7.55E-06 |
| QRS | 11 | rs113652043 | 25221599 | C | A | 6.13E-06 |
| QRS | 11 | rs74329445 | 45550368 | A | G | 7.58E-06 |
| QRS | 11 | rs74360883 | 45550753 | A | G | 7.51E-06 |
| QRS | 11 | rs77406754 | 60041989 | G | A | 8.98E-07 |
| QRS | 11 | rs76809954 | 83580306 | T | C | 1.32E-06 |
| QRS | 11 | rs61902539 | 1.13E+08 | G | T | 5.20E-06 |
| QRS | 12 | rs7297190 | 1987044 | T | C | 5.02E-06 |
| QRS | 12 | rs7308134 | 1987245 | G | A | 3.28E-06 |
| QRS | 12 | rs58041839 | 1988524 | G | A | 7.57E-06 |
| QRS | 12 | rs60945277 | 1989018 | T | C | 7.86E-06 |
| QRS | 12 | rs61085710 | 1989197 | A | G | 8.28E-06 |
| QRS | 12 | rs11838338 | 1989355 | G | A | 7.73E-06 |
| QRS | 12 | rs11829061 | 1989584 | G | C | 7.77E-06 |
| QRS | 12 | chr12:17728980:D | 17728980 | AT | A | 9.27E-06 |
| QRS | 12 | rs1186300 | 17728991 | C | T | 8.93E-06 |
| QRS | 12 | rs7964992 | 19016296 | T | A | 2.65E-06 |
| QRS | 12 | rs10437758 | 19018632 | C | T | 2.84E-06 |
| QRS | 12 | rs1490734 | 19019467 | T | A | 6.24E-06 |
| QRS | 12 | rs12313606 | 19021912 | G | A | 3.46E-06 |
| QRS | 12 | rs1844687 | 19023236 | G | A | 2.28E-06 |
| QRS | 12 | rs12319113 | 19024558 | G | A | 4.88E-07 |
| QRS | 12 | rs11836240 | 87136991 | G | T | 7.15E-06 |
| QRS | 12 | rs11104067 | 87141799 | T | A | 6.92E-06 |
| QRS | 12 | rs2195233 | 87142419 | A | C | 6.86E-06 |
| QRS | 12 | rs2217235 | 87145223 | A | G | 5.11E-06 |
| QRS | 13 | rs6490455 | 30961468 | G | C | 8.92E-07 |
| QRS | 14 | rs2274511 | 33829320 | C | T | 8.40E-07 |
| QRS | 14 | rs76523308 | 45045390 | C | T | 1.65E-06 |
| QRS | 14 | rs79842714 | 45045798 | G | T | 6.89E-07 |
| QRS | 14 | rs113317269 | 45046629 | C | T | 6.49E-07 |
| QRS | 14 | rs80035764 | 45046654 | C | T | 6.49E-07 |
| QRS | 14 | rs78526838 | 45047524 | C | T | 9.54E-07 |
| QRS | 14 | chr14:45047682:D | 45047682 | TG | T | 9.52E-07 |
| QRS | 14 | rs78553409 | 45048218 | C | T | 9.67E-07 |
| QRS | 14 | rs79983672 | 45048781 | T | C | 9.75E-07 |
| QRS | 14 | rs114431653 | 45049517 | G | A | 9.85E-07 |
| QRS | 14 | rs112685649 | 45049594 | C | A | 9.88E-07 |
| QRS | 14 | rs149468542 | 45049884 | T | G | 9.93E-07 |
| QRS | 14 | rs140090782 | 45050584 | T | C | 1.02E-06 |
| QRS | 14 | rs141081227 | 45051231 | T | C | 1.02E-06 |
| QRS | 15 | rs1356410 | 42434837 | T | C | 3.07E-06 |
| QRS | 15 | rs57017013 | 63891961 | G | A | 1.69E-06 |
| QRS | 16 | rs4781415 | 13217905 | A | G | 8.49E-06 |
| QRS | 16 | rs7184362 | 55879620 | A | G | 2.85E-06 |
| QRS | 18 | rs8093266 | 1800338 | G | A | 7.52E-06 |
| QRS | 18 | rs8091249 | 1802443 | T | G | 7.31E-06 |
| QRS | 18 | rs7241970 | 1803913 | T | C | 7.53E-06 |
| QRS | 18 | rs7227078 | 1804120 | T | C | 7.52E-06 |
| QRS | 18 | rs7227080 | 1804124 | T | G | 7.51E-06 |
| QRS | 18 | rs62079387 | 1805200 | T | C | 7.15E-06 |
| QRS | 18 | rs11660591 | 1805225 | T | C | 8.16E-06 |
| QRS | 18 | rs9964595 | 1807674 | G | A | 4.16E-06 |
| QRS | 19 | rs72974768 | 3435545 | G | A | 2.60E-06 |
| Phenotype | Chromosome | rsid | pos | allele_A | allele_B | QTC_430_frequentist_add_expected_pvalue |
| QTC430 | 1 | rs150968551 | 47428558 | T | G | 3.96E-07 |
| QTC430 | 1 | rs3762318 | 67597119 | G | A | 1.24E-06 |
| QTC430 | 1 | rs2024825 | 67597977 | T | C | 1.86E-06 |
| QTC430 | 1 | chr1:67600009:D | 67600009 | AC | A | 1.01E-06 |
| QTC430 | 1 | rs12069782 | 67600101 | C | T | 3.26E-07 |
| QTC430 | 1 | rs12095536 | 67600142 | T | C | 1.41E-06 |
| QTC430 | 1 | rs12090164 | 67600163 | G | A | 8.76E-06 |
| QTC430 | 2 | rs34198350 | 48169382 | C | T | 7.98E-06 |
| QTC430 | 2 | rs12623744 | 1.85E+08 | T | A | 9.03E-06 |
| QTC430 | 2 | rs11691711 | 1.85E+08 | G | A | 1.78E-06 |
| QTC430 | 2 | rs12472209 | 1.85E+08 | G | T | 5.46E-06 |
| QTC430 | 3 | rs11917787 | 253919 | A | G | 9.27E-06 |
| QTC430 | 3 | rs7645841 | 1.43E+08 | C | A | 5.09E-06 |
| QTC430 | 6 | rs12195574 | 13225971 | G | A | 4.44E-06 |
| QTC430 | 6 | rs116624347 | 30224238 | G | T | 3.79E-06 |
| QTC430 | 6 | rs115836987 | 30227399 | A | G | 4.33E-06 |
| QTC430 | 7 | rs10226069 | 68025176 | T | A | 9.69E-06 |
| QTC430 | 7 | rs10266640 | 68025239 | C | T | 9.96E-06 |
| QTC430 | 7 | rs10282048 | 68025407 | A | G | 7.88E-06 |
| QTC430 | 7 | rs10240115 | 68025960 | G | A | 7.81E-06 |
| QTC430 | 7 | rs13236930 | 68026083 | C | G | 7.38E-06 |
| QTC430 | 7 | rs2692877 | 1.09E+08 | G | A | 5.05E-06 |
| QTC430 | 7 | rs10276075 | 1.09E+08 | C | T | 6.39E-06 |
| QTC430 | 10 | rs61458523 | 60668571 | G | A | 1.96E-06 |
| QTC430 | 10 | rs11198013 | 1.19E+08 | C | T | 3.79E-06 |
| QTC430 | 12 | chr12:17638265:I | 17638265 | A | AAAAC | 1.99E-06 |
| QTC430 | 12 | rs10840728 | 17638662 | T | C | 9.16E-06 |
| QTC430 | 12 | rs10219728 | 17639821 | A | G | 6.46E-06 |
| QTC430 | 12 | rs10219673 | 17639951 | T | A | 6.31E-06 |
| QTC430 | 12 | rs1706605 | 17644613 | G | T | 9.96E-06 |
| QTC430 | 16 | chr16:79485899:I | 79485899 | T | TG | 7.57E-06 |
| QTC430 | 17 | rs116908816 | 32422699 | G | T | 8.94E-06 |
